# Redox-metabolic circuits as a central regulator of T cell-based immunotherapy

**DOI:** 10.3389/fimmu.2026.1807087

**Published:** 2026-05-14

**Authors:** Sarah McPhedran, Tian Zhao, Julian J. Lum

**Affiliations:** 1 Trev and Joyce Deeley Research Centre, BC Cancer, Victoria, BC, Canada; 2 Basic and Translational Research, BC Cancer Research Institute, Vancouver, BC, Canada; 3 Department of Biochemistry and Microbiology, University of Victoria, Victoria, BC, Canada

**Keywords:** GCN2, immunometabolism, immunotherapy, methionine, redox, T cells

## Abstract

T cell–based immunotherapies have transformed cancer treatment, yet their efficacy in solid tumors is constrained by the nutrient-poor and oxidative tumor microenvironment (TME). Accumulating evidence indicates that reactive oxygen species (ROS), methionine metabolism, and the amino acid stress sensor general control nonderepressible 2 (GCN2) are tightly interconnected regulators of T cell activation, differentiation, and effector function. In this review, we detail how these pathways form an integrated redox–metabolic circuit that dynamically tunes T cell responses to environmental stress. Physiological ROS are essential for T cell receptor signaling, glycolytic reprogramming, and cytotoxicity, whereas excessive or prolonged oxidative stress drives exhaustion and apoptosis. GCN2 links amino acid availability, particularly methionine and cysteine, to adaptive transcriptional and metabolic programs that regulate glutathione synthesis and redox homeostasis. We highlight how therapeutic manipulation of methionine availability, GCN2 signaling and ROS produces highly context-dependent outcomes across immune checkpoint blockade and adoptive cell therapy settings in solid tumors. Finally, we discuss emerging strategies to interrogate and modulate this circuit using integrated omics, CRISPR-based screening, and pharmacological approaches, emphasizing the need for context-aware and temporally controlled metabolic interventions to enhance T cell–based immunotherapies in solid tumors.

## Introduction

Metabolism has emerged as a central driver of T cell anti-tumor immunity. It is well established that T cells shift their metabolism based on their differentiation state and functional capacity: namely, T naïve (Tn) and T memory (Tm) cells mainly rely on oxidative phosphorylation (OXPHOS) for their bioenergetic needs, whereas T effector (Te) cells, which are dominantly responsible for tumor killing, are fueled by glycolysis (1–3). Similarly, exhausted T cells display reduced glycolytic flux and spare respiratory capacity (4). This knowledge has laid a foundation for modulating T cell metabolism as a strategy to augment therapeutic need, such as strengthening immunotherapies to treat solid tumors. For example, T cell-based cancer immunotherapy is now being looked at through the lens of metabolic engineering, conditioning and pharmacological intervention (5–10).

Redox metabolism, methionine metabolism, and the general control nonderepressible 2 (GCN2) pathway have all garnered increasing attention in the cancer immunotherapy field as potential therapeutic targets. This review will highlight the underappreciated connection between GCN2, methionine metabolism, and the transsulfuration pathway, and how these pathways collectively tune the redox state of T cells. We will discuss these pathways as components of an integrated metabolic circuit that is essential for T cell function and that can be manipulated to enhance cancer immunotherapy.

## Sources of ROS in T cells

Redox metabolism encompasses the cellular oxidation–reduction reactions that occur within cells, often mediated by redox cofactors such as NAD^+^/NADH, as well as the production of reactive oxygen species (ROS) and the antioxidant systems that regulate and eliminate ROS (11). The primary focus of this review will be on the production and downstream functions of ROS, however, other components of redox metabolism will be briefly discussed.

ROS are a byproduct of metabolic pathways in T cells, while also playing an indispensable role in T cell biology. The primary contributors to ROS production reside in the mitochondria, which supply over 90% of cellular ROS (12). The electron transport chain (ETC), a component of OXPHOS and the dominant producer of adenosine triphosphate (ATP), is located in the inner mitochondrial membrane. During the electron transport, superoxide (O_2_^-^) is generated through reactions catalyzed by complex I, II and III (12). Most superoxide is rapidly converted into hydrogen peroxide (H_2_O_2_), which can freely diffuse into the cytosol (12, 13).

Another important source of ROS in T cells is the family of nicotinamide adenine dinucleotide phosphate (NADPH) oxidases, including NOX2 and DUOX2. These enzymes play a role in maintaining redox cycling during the many metabolic reactions activated upon T cell receptor (TCR) stimulation (14). NOX2 produces superoxide as a byproduct of NADPH oxidation to NADP^+^ at the plasma membrane (15).

The endoplasmic reticulum (ER) also contributes to ROS production in T cells (16). Proteins that contain a disulfide bond, such as the TCR, produce hydrogen peroxide as a byproduct of disulfide bond formation. This process occurs through the oxidation of protein disulfide isomerase (PDI), a molecular chaperone critical for disulfide bond formation and rearrangement, which transfers electrons to molecular oxygen during catalysis (17). Furthermore, the ER indirectly influences ROS through calcium (Ca^2+^) signalling upon TCR stimulation (18). Calcium influx into mitochondria enhances ETC activity, thereby increasing mitochondrial ROS (mtROS) generation (18). Another indirect contribution of the ER to ROS arises during protein overload, when the ER reaches its folding capacity and activates the unfolded protein response (UPR). Although the UPR ultimately induces antioxidant programs, it initially increases ROS levels through the activation of stress response pathways (19, 20).

Finally, a recurring theme in this review is amino acid deprivation as a stimulator of ROS accumulation. Amino acid limitation can indirectly increase ROS through dysregulated mitochondrial dynamics and ER stress. This review, however, will primarily focus on glutathione (GSH) depletion as a central mechanism of ROS dysregulation. GSH is the primary intracellular redox buffer in T cells and plays an essential role in supporting anti-tumor responses (21). Deprivation of amino acids required for GSH synthesis, particularly methionine, cysteine, and glycine limits GSH production, leaving T cells unable to adequately buffer ROS (22).

Overall, multiple pathways in T cells indirectly or directly contribute to ROS production, including ones not mentioned here (e.g. ROS produced in the peroxisome), with the mitochondrial ETC being the dominant means of ROS production.

## Measuring cellular ROS

The simplest and most widely used methods to detect ROS rely on fluorescent dyes that can be quantified by flow cytometry or microscopy. Common examples include 2’,7’-dichlorodihydrofluorescein diacetate (DCF), MitoSOX Red, and dihydroethidium (DHE) (23). Additionally, ROS can also be assessed using genetically encoded sensors, such as roGFP2-based probes or members of the HyPer family, which allow for more targeted and dynamic measurements (23). In addition to these direct approaches, ROS can be measured indirectly through downstream readouts, including the GSH/GSSG and NADPH/NAD^+^ ratios, often quantified using metabolomics-based techniques (23). Each of these methods has distinct advantages and limitations and is best suited to specific experimental contexts, as reviewed extensively by Murphy et al. (23). Refer to **Table 1** for a summary of methods to measure ROS.

**Table 1:** Methods of ROS detection.

Category	Method / probe	What it measures	Key advantages	Key limitations / notes
Fluorescent dyes (general ROS)	DCF (H2DCFDA)	Broad intracellular ROS (mainly H2O2-derived oxidation)	Simple, widely used, flow cytometry compatible	Non-specific; oxidation-dependent signal; prone to artifacts
	CellROX dyes	General oxidative stress	More stable than DCF; good for imaging	Still relatively non-specific
Superoxide-specific dyes	DHE	Cytosolic superoxide (O2•−)	Common, relatively sensitive	Can form multiple oxidation products; requires careful interpretation
	MitoSOX Red	Mitochondrial superoxide	Mitochondria-targeted; useful for metabolic studies	Can be oxidized non-specifically at high ROS
Genetically encoded sensors	roGFP2 (± Grx1, Orp1)	Redox potential / H2O2 (depending on fusion)	Ratiometric, reversible, quantitative	Requires transduction; lower throughput
	HyPer family	H2O2 specifically	High specificity, real-time dynamics	pH sensitivity; needs controls
Indirect metabolic readouts	GSH/GSSG ratio	Cellular redox buffering capacity	Quantitative, biologically meaningful	Indirect; does not measure ROS species directly
	NADPH/NADP^+^ ratio	Redox metabolism / antioxidant capacity	Links to metabolic state	Indirect; influenced by many pathways
Mass spectrometry / metabolomics	Untargeted or targeted metabolomics	Redox metabolites (e.g., glutathione, cysteine)	High depth, systems-level insight	Expensive; indirect
Protein oxidation markers	Protein carbonylation, sulfenylation (e.g., dimedone-based probes)	Oxidative damage to proteins	Biologically relevant readout	Endpoint measurement; not dynamic
Lipid peroxidation assays	MDA, 4-HNE	Oxidative damage to lipids	Clinically relevant biomarkers	Indirect; reflects accumulated damage
DNA oxidation	8-oxo-dG	Oxidative DNA damage	Useful for stress/damage studies	Not a real-time ROS measure
Electron spin resonance (ESR/EPR)	Spin trapping of free radicals	Direct detection of specific ROS species	Gold standard, highly specific	Technically demanding, low throughput
Mitochondrial function assays	Seahorse (OCR/ECAR)	Indirect ROS-linked metabolic stress	Functional metabolic context	Does not measure ROS directly

## Antioxidant pathways in T cells

Given the multitude of metabolic pathways that generate ROS in T cells, robust antioxidant systems are required to prevent ROS accumulation beyond levels compatible with optimal T cell function. As noted above, the primary antioxidant hub in T cells is the GSH pathway, which is readily available in the cytosol during periods of elevated ROS production, such as TCR stimulation (24). GSH is highly nucleophilic, making it an effective scavenger of ROS. With catalytic assistance from glutathione peroxidases (GPXs), GSH reduces a wide range of ROS to water (25, 26).

Another important antioxidant system in T cells is the peroxiredoxin (PRDX) family. PRDX enzymes primarily regulate ROS generated during TCR signalling, rather than ROS produced during overt stress responses. There are six PRDX isoforms, each of which plays distinct roles in T cells (27–29). PRDX1 and PRDX2 are cytosolic and contribute to ROS regulation. However, PRDX1 additionally participates in cellular signaling, whereas PRDX2 functions predominantly as an antioxidant (30). PRDX3 and PRDX4 scavenge ROS within the mitochondria and ER, respectively, while PRDX5 and PRDX6 localize to multiple cellular compartments (29).

Additional detoxification systems include the thioredoxin (TRX1/2) pathway, which is particularly important for neutralizing ROS generated during disulfide bond formation in the ER, and superoxide dismutase 2 (SOD2), which catalyzes the conversion of superoxide into hydrogen peroxide (31, 32). Beyond enzymes that directly detoxify ROS, several metabolic pathways indirectly support antioxidant capacity. For example, the pentose phosphate pathway (PPP) regenerates NADPH, which is required to maintain GSH in its reduced state (33). Similarly, the transcription factor NRF2 is activated under oxidative stress and drives the expression of numerous antioxidant genes, including those encoding PRDXs and enzymes involved in GSH metabolism (34).

## ROS: a careful balance

**Figure 1:**
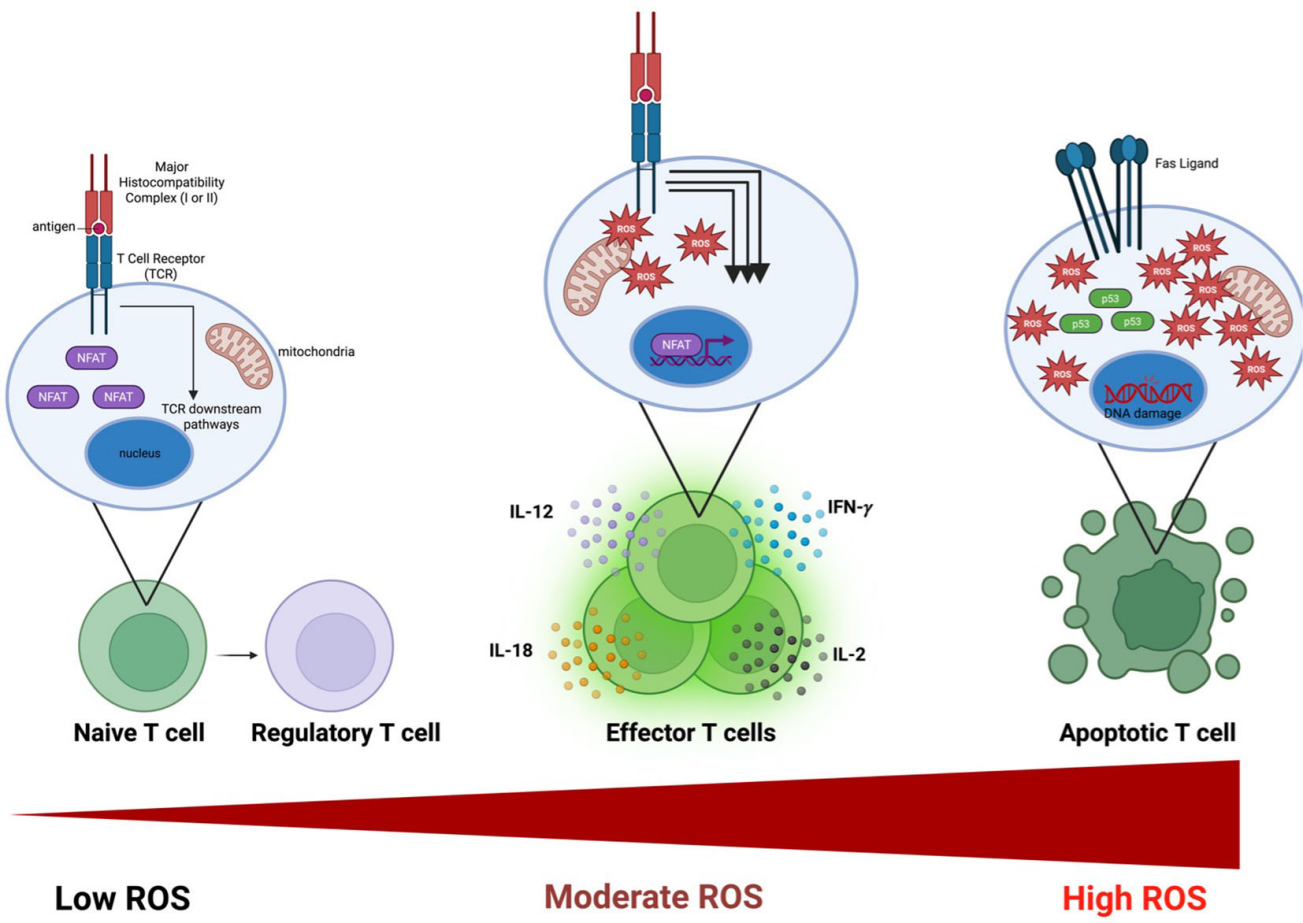
Reactive oxygen species (ROS) exert dose-dependent control over T cell fate and function. Low intracellular ROS levels during antigen recognition favor weak T cell receptor (TCR) signaling (i.e. Lck->ZAP-70->LAT->->downstream LAT targets) and support regulatory T cell (Treg) differentiation (i.e. stabilizing FOXP3). Moderate, transient increases in ROS following TCR engagement promote mitochondrial signaling, sustained NFAT activation, and downstream metabolic reprogramming (e.g. enhanced glycolysis), driving effector T cell differentiation and cytokine production (e.g., IFN-γ, IL-2, IL-12, IL-18). In contrast, excessive or prolonged ROS accumulation results in oxidative damage, activation of stress and apoptotic pathways (including p53 and Fas–FasL signaling), mitochondrial dysfunction, and DNA damage, ultimately leading to T cell exhaustion or apoptosis. Together, these states illustrate a “Goldilocks” model in which ROS must be tightly tuned to support optimal anti-tumor T cell immunity.

Maintenance of ROS homeostasis is critical across all cell types, and T cells are no exception. ROS levels increase dramatically upon TCR stimulation, yet ROS are not merely metabolic byproducts. Instead, ROS produced by T cells and surrounding immune cells are essential for TCR signaling and sustained effector function. Conversely, excessive ROS accumulation leads to DNA damage, induction of p53 and Fas ligand (FasL), and ultimately T cell apoptosis (**Figure 1**), which can be advantageous in terms of controlling autoimmunity (35). However, in environments such as the tumor microenvironment [e.g. pancreatic ductal adenocarcinoma (PDAC), lung cancer, melanoma, etc.], excessive ROS can cause high T cell dysfunction and death (36–39). Many reviews have covered the topic of ROS balance in T cells extensively (40–42).

Given this finite balance, it is not surprising that ROS are generated by multiple pathways following TCR activation and, in parallel, eliminated by multiple antioxidant systems. This redundancy is essential to maintain ROS at concentrations that support, rather than impair, T cell function.

## What is the GCN2 pathway?

General Control Non-derepressible-2 (GCN2) is a translational and metabolic regulator that coordinates cellular processes in response to stress, including amino acid deprivation (43). Upon depletion of amino acids, GCN2 binds to uncharged tRNAs – tRNAs not attached to amino acids – and becomes activated through autophosphorylation (44). Activated GCN2 phosphorylates Eukaryotic Translation Initiation Factor 2α (eIF2α), resulting in a global reduction in translation while selectively enhancing the translation of mRNAs containing upstream open reading frames (uORFs), including Activating Transcription Factor 4 (ATF4) mRNA (45–50). The GCN2-ATF4 pathway subsequently induces the expression of genes involved in amino acid transportation and metabolism (51, 52). In addition, this pathway also promotes survival through AKT activation, suppression of mTORC1 and induction of autophagy (53–56), the latter includes mitophagy (57), which plays a key role in limiting excess ROS production by removing damaged mitochondria (58). The GCN2-ATF4 pathway also modulates redox homeostasis by inducing the expression of Glutathione peroxidase 1 (GPX1), a key antioxidant enzyme (59). Moreover, the pathway has also been shown to activate the NRF2 signaling pathway in macrophages in response to leucine depletion (60).

The GCN2-ATF4 axis further regulates redox balance by upregulating amino acid transporters, including SLC1A4, SLC7A5/SLC3A2 (LAT1), SLC7A11 (xCT), and SLC1A5 (ASCT2), thereby increasing uptake of methionine and cysteine, among other amino acids (51, 52). Both methionine and cysteine contribute to GSH biosynthesis. In addition, in response to cysteine depletion, the GCN2-ATF4 pathway maintains redox homeostasis by inducing enzymes of the transsulfuration pathway, which synthesizes cysteine from methionine in a methionine cycle-dependent manner (61). Thus, methionine and cysteine connect nutrient sensing to ROS regulation through GCN2 signaling and GSH synthesis.

## Methionine and cysteine fuel GSH synthesis through the methionine cycle and the transsulfuration pathway

GSH is synthesized through two energy-dependent steps. The first, rate-limiting step involves the ligation of cysteine to glutamate to form γ-glutamylcysteine, catalyzed by γ-glutamylcysteine ligase (GCL). The second step is mediated by glutathione synthase (GS), which conjugates γ-glutamylcysteine with glutamine to generate GSH (22). This process is primarily limited by the availability of cysteine (62).

T cells acquire cysteine through the transporters SLC1A4 and SLC1A5, as well as the cystine (oxidized cysteine) transporter, xCT (63). Alternatively, T cells can acquire cysteine *de novo* synthesis by converting methionine to cysteine through the methionine cycle and the transsulfuration pathway, a process that is crucial for promoting T cell survival under oxidative stress (64). In the methionine cycle, methionine is first converted to homocysteine, losing a methyl group in the process (65). Homocysteine is then ligated with serine by cystathionine β-synthase (CBS) to form cystathionine, which is subsequently cleaved by cystathionine γ-lyase (CTH) to generate cysteine (61).

Beyond its role in fueling GSH synthesis, the methionine cycle also produces metabolites essential for multiple crucial cellular functions. One of such metabolites, S-adenosylmethionine (SAM), serves as a universal methyl group donor required for proteins, RNAs, and DNA methylation. SAM shapes T cell activation, differentiation, and antitumor function through epigenetic and posttranscriptional modifications (66–70). The role of SAM in epigenetic regulation of T cell activation and effector function has been reviewed previously (65). Additionally, SAM is required for *de novo* polyamine synthesis, which governs the fidelity of T cell differentiation and can suppress T cell anti-tumor function (71–74). Besides SAM, other metabolites derived from the methionine cycle also contribute to the regulation of T cell responses, as reviewed previously (65).

## Section 1: importance of ROS, GCN2, and methionine pathways in regulating T cell anti-tumor immunity

### Importance of ROS in T cells

Reactive oxygen species (ROS) play an essential role in T cell activation, subset formation, and effector function. The most direct role that ROS play in T cell function occurs during activation. Once the T cell receptor (TCR) is stimulated along with a costimulatory signal from an antigen presenting cell (APC), mitochondrial electron flux is increased. This results in a substantial buildup of hydrogen peroxide in the cytosol, which works to reversibly oxidize catalytic cysteine residues in phosphatases, ultimately preventing them from dephosphorylating key downstream TCR signaling proteins, including ZAP-70, PTEN, and LAT (75, 76). Importantly, ROS-mediated signaling during T cell activation occurs within a tightly regulated spatial and temporal window, as excessive diffusion of hydrogen peroxide would otherwise oxidize iron–sulfur cluster–containing electron transport chain (ETC) components and impair mitochondrial function. Activation of TCR signalling proteins leads to robust signal amplification that drives the imminent effector phenotype of the T cell. Similarly, ROS enhance calcium signaling by sustaining store-operated calcium entry (SOCE) activity, thereby prolonging calcium flux into the cytosol (77). Calcium signaling plays many important roles in T cell activation; notably, it is essential for the translocation of Nuclear Factor of Activated T cells (NFAT) into the nucleus (**Figure 1**) (78). Furthermore, ROS play a multitude of additional roles in initiating the T cell response, such as activating key transcription factors (e.g., NF-κB, AP-1) and the mechanistic target of rapamycin complex 1 (mTORC1), the master regulator of cellular metabolism (79).

ROS also play a critical role in delineating T cell subset formation and, reciprocally, cytokines, the messengers of the immune system, greatly influence ROS. As a byproduct of amplified TCR activation, T cells shift toward an effector phenotype, the key subset responsible for tumor killing (80, 81). In CD4 T cells, this ROS-induced effector phenotype is characterized by a bias toward T helper 1 (Th1) cells, the dominant anti-tumor CD4 subset, as well as Th17 cells (13, 82). Conversely, this phenotype is reversed if high levels of ROS linger after activation, in which case a Th2 phenotype is promoted (83). Likewise, low ROS levels during initial activation can favor regulatory T cell (Treg) formation by promoting a shift toward oxidative phosphorylation (OXPHOS) and stabilizing FOXP3 (82, 84). Furthermore, antioxidant defenses are essential for maintaining Treg suppressive capacity, and treatment with N-acetyl cysteine (NAC), an ROS scavenger, can reverse Treg dysfunction induced by elevated ROS levels (84, 85). When T cell activation is sustained for prolonged periods, such as within the tumor microenvironment, elevated cellular ROS can push T cells toward an exhausted phenotype due to damage to DNA and mitochondria (86, 87). Following the initial ROS spike after activation, intracellular ROS levels decline. Memory T cell formation depends on moderate levels of mitochondrial ROS generation, originating from complex III (88). Together, these findings highlight the “Goldilocks” role of ROS: too little fails to induce effector T cell formation and instead promotes Tregs, whereas excessive or prolonged ROS drives exhaustion and apoptosis.

T cell–mediated tumor killing relies on cells that adopt a Warburg-like metabolic phenotype characterized by high glycolytic flux (89–91). Strong evidence suggests that ROS play an important role in amplifying and maintaining a T cell glycolytic phenotype through multiple mechanisms. Primarily, ROS sustain TCR signaling via PTEN inactivation, activating mTORC1 and AKT to promote the translation and stabilization of glycolytic enzymes (92–95). In addition, ROS stabilize and enhance hypoxia-inducible factor-1α (HIF-1α), the oxygen-sensitive subunit of the HIF-1 transcription factor, by inhibiting prolyl hydroxylase–mediated degradation under both hypoxic and inflammatory conditions. Stabilized HIF-1α translocate to the nucleus, where it dimerizes with HIF-1β and drives the transcription of genes that promote glycolytic metabolism, including glucose transporters and key glycolytic enzymes such as GLUT1 and lactate dehydrogenase A (LDHA), thereby reinforcing a metabolic shift toward aerobic glycolysis (94–96). Similarly, ROS play a critical role in NFAT activation, which promotes the transcription of genes that support glycolysis. NFAT induces expression of genes such as MYC, a key regulator of glycolytic metabolism, and IL-2, which signals through the PI3K–AKT–mTOR pathway to enhance glycolytic activity (13, 97). In addition, NFAT can increase chromatin accessibility by recruiting the histone acetyltransferases CBP/p300, leading to H3K27 acetylation and facilitating the binding of glycolysis-promoting transcription factors, including HIF-1α (98, 99). Collectively, ROS-supported signaling and transcription factor driven glycolytic programming promote the secretion of effector molecules, including IFN-γ and perforin/granzyme, that are essential for T cell function.

Taken together, ROS play an indispensable role in promoting anti-tumor immunity by sustaining T cell activation, biasing differentiation away from a Treg phenotype, and promoting the glycolytic metabolism required for T cell cytotoxicity. Importantly, ROS enhance T cell–mediated anti-tumor immunity only within a low to moderate range, whereas excessive ROS drive T cell exhaustion and apoptosis.

### GCN2 regulates T cell anti-tumor function by sensing amino acid depletion

Not only are amino acids building blocks for protein synthesis, but they also fuel metabolic pathways to support T cell proliferation and function. For example, methionine supports epigenetic reprogramming during T cell differentiation by generating the methyl group donor SAM (66, 67). Cystine, cysteine and methionine are essential for maintaining redox balance during T cell activation, as they are required for GSH synthesis (64, 100). Serine and glycine serve as key precursors for purine synthesis in T cells, enabling proliferation upon activation, while glutamine fuels the tricarboxylic acid (TCA) cycle to support ATP and pyrimidine synthesis during T cell activation (101, 102). In addition to these metabolic roles, several amino acids directly regulate T cell anti-tumor immunity. Arginine promotes oxidative phosphorylation and drives T cell memory formation, thereby enhancing anti-tumor function (103). The branched-chain amino acids, leucine, isoleucine, and valine, enhance glucose metabolism through the activation of mTOR, leading to improved anti-tumor function of T cells (100, 101). In addition, tryptophan depletion arrests T proliferation and sensitizes T cells to apoptosis (104). Moreover, the catabolism of tryptophan into kynurenine by indoleamine 2,3-dioxygenase (IDO) downregulates TCR ζ-chain expression and promotes the differentiation of regulatory T cells, contributing to immunosuppression (105).

However, the tumor microenvironment is often nutrient-poor, including depletion of specific amino acids (106). For example, arginine, cystine, and tryptophan are depleted in the interstitial fluid of pancreatic ductal adenocarcinoma (PDAC) (107). Similarly, glutamine, glutamate, and arginine levels were downregulated in melanoma interstitial fluid (108). As such, a mechanism to sense and respond to amino acid depletion may be crucial for T cell anti-tumor response in solid tumors.

One such mechanism is the GCN2 pathway. Early studies on the role of GCN2 in T cells have primarily focused on its role in IDO-mediated immune suppression. IDO converts tryptophan into the immunosuppressive metabolites kynurenine, resulting in tryptophan depletion and kynurenine accumulation – an immune tolerance mechanism often hijacked by tumors. These studies demonstrated that GCN2-deficient T cells are resistant to IDO-mediated proliferation arrest and TCR ζ-chain downregulation, suggesting that GCN2 is crucial for IDO-mediated immune suppression (105, 109). However, GCN2 is not required for T cell proliferation arrest induced by tryptophan depletion alone. In contrast, subsequent studies showed that GCN2 upregulates stress response genes during T cell activation under amino acid-restricted conditions and is required for optimal T cell proliferation upon antigen stimulation, suggesting a supportive role for GCN2 in T cell activation in nutrient-restricted environments (110). Interestingly, one study reported that GCN2 is dispensable for T cell anti-tumor activities in murine melanoma models, as T cell-specific GCN2 knockout neither impairs T cell responses against tumors, nor does it significantly affect FoxP3 expression in CD4+ T cells or IFN-γ expression in both CD4+ and CD8+ T cells in the tumor. Notably, GCN2 was not activated in T cells in these tumors, likely due to sufficient intratumoral tryptophan levels (111). By contrast, a more recent study demonstrated that GCN2 is crucial for CD8+ T cell anti-tumor function in murine glioma models exhibiting IDO activities. In this setting, knocking out GCN2 reduced CD8+ T cell survival and protein kinase C θ (PKCθ) phosphorylation under low-tryptophan conditions *in vitro*, and GCN2 knockout leads to decreased CD8+ T cell numbers, and reduced CD44 and granzyme B expression in CD8+ T cells within the tumor, resulting in reduced survival (112). Consistent with these findings, treatment of activated CD8+ T cells ex vivo with a GCN2 agonist halofuginone (halo) increased IFN-γ and Granzyme B expression. In addition, halo-treated OTI T cells showed enhanced anti-tumor function in murine lymphoma models, leading to reduced tumor growth and improved survival. Mechanistically, halo augments T cell effector function by increasing oxidative phosphorylation and activating the CD98 (SLC7A5/SLC3A2)-mTOR axis. Interestingly, halo-treated CD8+ T cells also showed elevated intracellular methionine and glutathione (GSH) levels, suggesting enhanced antioxidant response induced by GCN2 (113). Whether this increased antioxidative response directly contributes to improved effector function remains to be determined.

### Role of methionine in T cell anti-tumor function

Studies examining the role of methionine in regulating T cell anti-tumor function have yielded conflicting findings. In cases where tumor cells outcompete T cells for methionine, methionine depletion reduces levels of the histone methylation mark H3K79me2, which decreases the expression of signal transducer and activator of transcription 5 (STAT5) in endogenous CD8+ T cells. Consequently, expression of IL-2, TNF-α, and IFN-γ was reduced, leading to impaired anti-tumor function (68). Additionally, reduced H3K79me2 levels caused by tumor-driven methionine depletion also increases the expression of inhibitory receptors such as programmed cell death protein 1 (PD-1) in endogenous CD4+ T cells, further suppressing T cell function (69). Restoring methionine availability in the tumor microenvironment rescues T cell function and improves tumor burden control, suggesting that methionine supports the anti-tumor function of endogenous T cells (69). Consistent with these findings, a recent study demonstrated that methionine restriction during early T cell activation induces T cell exhaustion by inhibiting methylation of the potassium channel KCa3.1, resulting in increased Ca^2+^ influx and excessive activation of NFAT (70).

Conversely, studies examining the effects of methionine in the context of immune checkpoint blockade (ICB) therapies indicate a more suppressive role. For example, dietary methionine restriction (MR) in combination with PD-1 blockade increases the abundance of tumor-infiltrating CD8+ T cells and enhances granzyme B expression. Crucially, the increase in CD8+ T cell effector function is associated with reduced presence of the immunosuppressive M2 macrophages in the tumor, which may result from suppressed M2 polarization driven by increased ROS production and nuclear factor of activated T cells 5 (NFAT5) expression under MR (114). These findings suggest that methionine indirectly suppress anti-tumor T cell function by promoting immunosuppressive macrophages. Consistent with this, Li et al. reported that dietary MR synergizes with PD-1 inhibition by reducing methylation of immune checkpoint mRNAs, including programmed death ligand 1 (PD-L1) and v-domain Ig suppressor of T cell activation (VISTA), in tumor cells, thereby enhancing CD8+ T cell infiltration and increasing granzyme B and IFN-γ expression (115). Similarly, a recent study showed that dietary MR enhances the efficacy of PD-1 blockade by increasing T cell number and granzyme B expression within tumors. Mechanistically, dietary MR was found to downregulate proprotein convertase subtilisin/kexin type 9 (PCSK9), a negative regulator of MHC-I expression and TCR signaling, through inhibition of DNA methylation (116). In line with these findings, two additional studies suggest a suppressive role for methionine in anti-tumor immunity in the context of ICB, but these studies did not examine T cells (117, 118). In contrast, one study reported that methionine supports T cell activation and anti-tumor immunity during ICB by supporting hydrogen sulfide production via the gut microbiota, indicating that methionine promotes T cell anti-tumor function in the presence of ICB (119). Consistently, supplementation of methionine through peritumoral injection reduces the expression of inhibitory histone methylation marks in CD8+ T cells and enhances the efficacy of ICB (70).

Beyond these conflicting observations, recent work has shown that intermittent dietary MR, rather than sustained dietary MR, accelerates tumor ferroptosis and enhances T cell infiltration and effector function. These findings suggest that the role of methionine in regulating T cell anti-tumor function is not only context-dependent but also time-dependent (120).

Lastly, although many studies have focused on methionine’s role in ICB, its impact on adoptive T cell therapies (ACT) has received considerably less attention. To address this gap, a recent study examined the effect of MR specifically on activated T cells, as T cells are activated and expanded *ex vivo* prior to infusion in ACT. Methionine was shown to play a time-dependent roles in regulating the effector function of activated T cells. Culturing activated T cells transiently in MR media inhibits synthesis of the immune suppressive polyamines, spermidine and spermine, leading to enhanced IFN-γ production and cytotoxicity against lymphoma cells *in vitro*. In contrast, prolonged exposure to MR *in vitro* induces expression of inhibitory receptors PD-1 and TIM-3. However, transient MR conditioning of CD8+ T cells prior to adoptive transfer into tumor-bearing mice does not improve anti-tumor efficacy, possibly because their function is reversed upon entering a methionine-replete environment. In contrast, sustained dietary MR reduces tumor control and overall survival, potentially due to increased inhibitory receptor expression, suggesting that continuous methionine availability is crucial for ACT efficacy (74). Consistent with this, Sharma et al. reported that daily peritumoral injection of methionine improves ACT efficacy in murine tumor models (70). Together, these findings suggest that methionine is required to support the anti-tumor function of adoptively transferred T cells.

### GCN2-Methionine-ROS metabolic circuit: role in tuning T cell function

**Figure 2:**
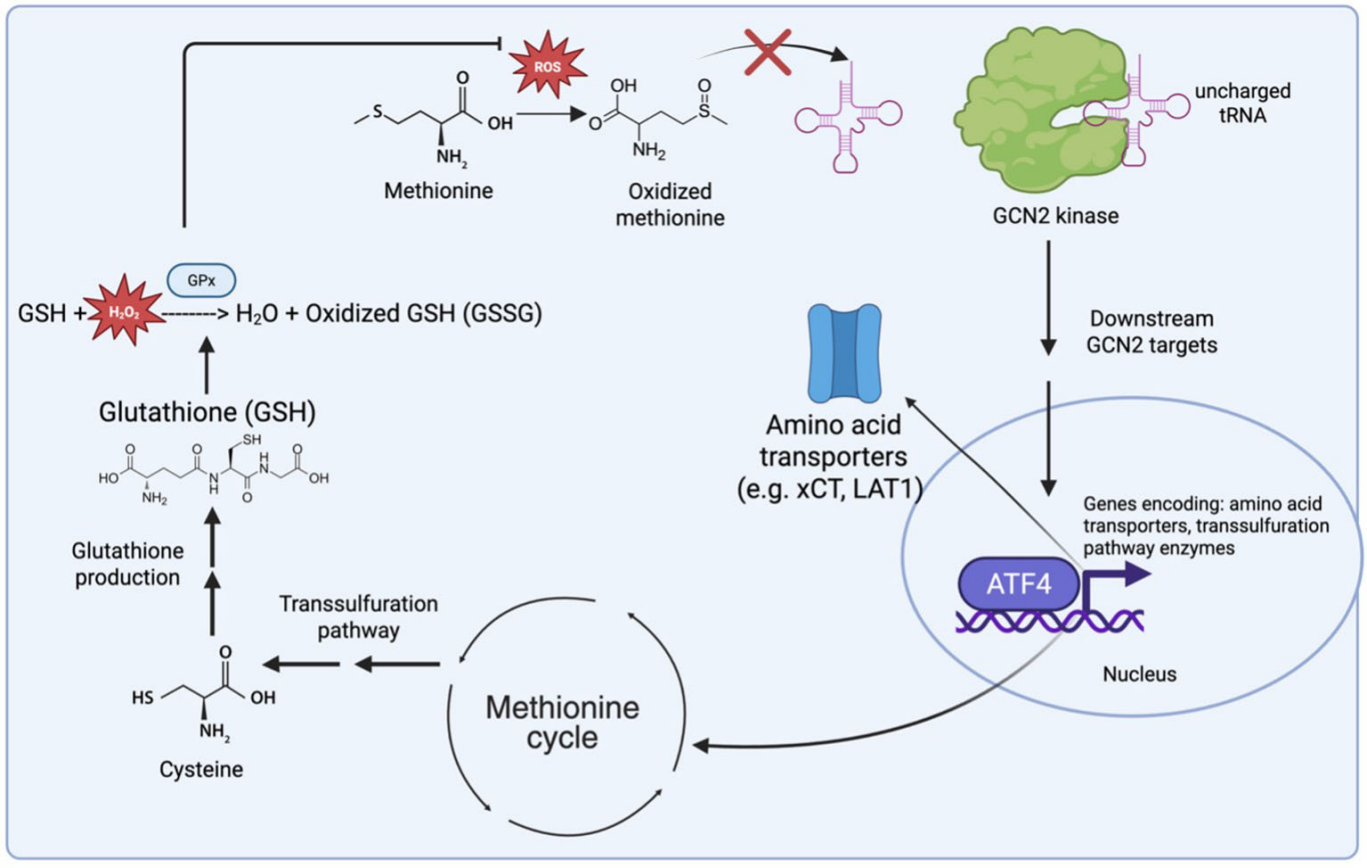
The GCN2–methionine–ROS metabolic circuit integrates amino acid sensing with redox homeostasis. Elevated reactive oxygen species (ROS) oxidize methionine, limiting its charging onto tRNAs and promoting accumulation of uncharged tRNAs that activate the amino acid stress sensor GCN2. Activated GCN2 signals through ATF4 to induce transcription of genes encoding amino acid transporters (e.g., xCT, LAT1) and enzymes of the methionine cycle and transsulfuration pathway. Transsulfuration-derived cysteine supports glutathione (GSH) synthesis, enabling detoxification of ROS through glutathione peroxidases (GPx) and restoring redox balance. This feedback circuit couples methionine availability, antioxidant capacity, and stress-responsive translational control, allowing T cells to adapt to oxidative and nutrient stress within the tumor microenvironment.

GCN2, methionine, and ROS each play independent yet integrated roles in tuning T cell responses to tumors, such that modulation of one pathway inevitably influences the others. Here, we outline how these pathways from a crucial metabolic circuit that is a key determinant of T cell function (**Figure 2**).

As mentioned, the primary trigger for GCN2 activation is amino acid deprivation, which results in the accumulation of uncharged tRNAs. High ROS environments can trigger amino acid deprivation in multiple ways, including oxidizing amino acids, such as methionine. MetO, the oxidized form of methionine, is a poor substrate for MetRS, the methionine-specific tRNA (44, 59, 121). GCN2 activation leads to downstream activation of ATF4, a central mediator of the integrated stress response (ISR) (50). ATF4 regulates a broad transcriptional program, including rerouting methionine into the methionine salvage and transsulfuration pathways. The transsulfuration pathway supports the synthesis of GSH, a critical antioxidant in T cells (61). In parallel, the ISR promotes expansion of intracellular amino acid pools, largely through the upregulation of amino acid transporters. Thus, two major outcomes of GCN2 activation are increased amino acid availability and reduced cellular ROS levels (52).

The GCN2–methionine–ROS circuit becomes particularly important when considering T cells within the tumor microenvironment (TME). In this context, GCN2 activity is elevated due to sustained environmental stress, including limited amino acid availability and high oxidative burden (109). This milieu can drive constitutive GCN2 activation, ultimately leading to reduced cellular ROS levels and the potential for impaired effector function (109). This phenomenon may play an integral role in dampening T cell activity within the TME. Therefore, modulation of the GCN2–methionine–ROS circuit may represent a promising strategy to enhance T cell–based immunotherapies for solid tumors.

## Section 2: therapeutic modulation

### Manipulating ROS to enhance T cell anti-tumor immunity

Given that reactive oxygen species (ROS) play multiple roles in priming T cells for a potent anti-tumor response, it is reasonable to suggest that modulating ROS may improve T-cell immunotherapies against solid tumors. Rather than operating as two mutually exclusive strategies, redox modulation could be designed to do both: engineering T cells to withstand chronic, damaging oxidative stress within the tumor microenvironment (TME) while preserving, or enhancing, transient, signaling-competent ROS required for effector function. This strategy becomes particularly important in solid tumors, where immunotherapy has shown limited success.

Several studies have demonstrated that disruption of the *NRF2* gene, which encodes a transcription factor responsible for inducing many antioxidant proteins, enhances T cell anti-tumor immunity. One study showed that NRF2 expression was increased in tumor-infiltrating lymphocytes (TILs) isolated from a hypoxic TME compared to control T cells (122). Likewise, IFN-γ production was dramatically increased in NRF2-deficient cells (122). Another study reported similar findings, showing that deletion of *Nrf2* in a murine adoptive T cell therapy (ACT) model enhanced tumor-killing capacity across several solid tumor models, including melanoma, lymphoma, colon carcinoma, and lung carcinoma (123). A separate group demonstrated that pre-treating T cells with auranofin prior to adoptive transfer, an Nrf2-activating compound, increased T cell effector function and tumor killing in a blood cancer model (124). These seemingly contradictory results suggest that NRF2 may play a context-dependent role in modulating T cell function, such as differences between solid and liquid tumors or between high- and low-oxidative-stress environments.

Similarly, one study showed that Tmed4(-/-) mice had endogenous regulatory T (Treg) cells that shifted toward a more inflammatory phenotype with enhanced anti-tumor activity in a colorectal cancer model (84). TMED4 is a potent activator of NRF2, and loss of TMED4 prevents NRF2 from inducing an antioxidant state within the cell. Indeed, the authors demonstrated that the increase in anti-tumor function resulting from *Tmed4* deletion was due to elevated ROS levels, which contributed to inhibition of FOXP3 (84). This work further supports the conclusion that abrogating NRF2 signaling in T cells can increase anti-tumor capacity against solid tumors.

Likewise, multiple groups have shown that increasing hydrogen peroxide levels in T cells can have beneficial effects on T cell function. One study demonstrated that Prdx2-/- mice, where PRDX2 is responsible for cytosolic hydrolysis of hydrogen peroxide, enhanced T cell function (125). In an lymphocytic choriomeningitis (LCMV) murine model, effector CD8^+^ T cells lacking *Prdx2* exhibited increased persistence and initial effector function, although this phenotype was reversed during chronic infection (125). While this effect was observed in a viral model, it suggests that loss of antioxidant capacity may confer an advantage in tissue environments, with implications for solid tumors. Another group showed that intratumoral injection of hydrogen peroxide alongside radiotherapy significantly reduced tumor growth in colon cancer and melanoma murine models (126). Importantly, this effect was partially T cell-dependent, as increased IFN-γ production and T cell persistence were observed in hydrogen peroxide-treated tumors (126). Together, these studies indicate that increasing cellular or environmental hydrogen peroxide can enhance T cell effector function.

It has been shown that conditioning T cells prior to ACT in a low-glucose environment primes them for increased effector function and persistence *in vivo*, leading to complete tumor clearance in a lymphoma murine model (8). One study sought to uncover the mechanism underlying this effect and found that glucose restriction alters the cellular redox state, ultimately increasing ROS production from mitochondrial complex III (127). Importantly, blunting mitochondrial ROS reversed the benefits conferred by glucose restriction prior to ACT (127). These findings lend further support to the idea that increasing ROS in specific contexts can enhance T cell anti-tumor immunity.

In the context of chimeric antigen receptor (CAR) T cell therapy, an adoptive cell ACT approach that engineers T cells to recognize tumour antigens, both enhancing and limiting antioxidant capacity have shown therapeutic promise. Specifically, overexpression of catalase in CAR-T cells improved tumour killing of an ovarian cancer cell line under oxidative stress, while *TRX1* overexpression enhanced pro-inflammatory cytokine production, proliferation, and cytotoxicity against a breast cancer cell line in oxidative conditions (31, 128). On the other hand, another study showed that NRF2 knockdown in human CAR-T cells enhanced anti-tumor immunity in a xenograft murine model implanted with a B-lymphoblastoid cell line, showing that eliminating the antioxidant effect may be beneficial in a lymphoma setting (124).

The seemingly contradictory findings regarding whether increasing or decreasing antioxidant capacity in T cells is beneficial warrant closer examination of the TME itself. It has long been known that the TME contains high levels of ROS, leading to the hypothesis that enhancing antioxidant capacity may help T cells retain function within this environment (129). However, multiple studies have demonstrated that oxidative stress within the TME is required for the efficacy of adoptive T cell therapy. One study showed that ROS accumulate in tumors following ACT and actively contribute to treatment efficacy (130). Furthermore, another study found that macrophage-dependent accumulation of peroxynitrite, a byproduct of superoxide, is triggered by T cell recognition of tumors and contributes to tumor cell cytotoxicity (131). Together, these findings suggest that ROS within the TME both directly enhance T cell anti-tumor immunity and cooperate with intratumoral immune cells to drive tumor cell killing.

In terms of clinical translation, several ROS-modulating agents already used in clinical settings warrant further investigation for their effects on T cell anti-tumor immunity. For example, N-acetyl cysteine (NAC), an ROS scavenger commonly taken as an over-the-counter supplement, has been shown in preclinical studies to enhance adoptive T cell therapy in melanoma (132, 133). Similarly, as discussed previously, auranofin has demonstrated the ability to enhance T cell anti-tumor immunity in preclinical models and holds strong potential for repurposing as a cancer therapeutic, given its established use in rheumatoid arthritis (134). Given that both compounds have well-characterized and tolerable safety profiles, it may be valuable to evaluate their effects on T cell function and anti-cancer efficacy in clinical studies (135, 136).

### Manipulating methionine to enhance T cell anti-tumor immunity

To date, many studies have sought to enhance anti-tumor responses in solid tumors by modulating methionine metabolism (68–70, 74, 114–118, 120). Collectively, these studies have suggested that methionine can exert both beneficial and suppressive effects on T cell responses in solid tumors. In the context of ACT or spontaneous anti-tumor response, methionine restriction (MR) has been shown to induce immune checkpoint receptor expression and T cell exhaustion, resulting in impaired anti-tumor T cell responses (69, 70, 74). In contrast, most studies examining methionine metabolism in the settings of immune checkpoint blockade (ICB) suggest that MR can enhance anti-tumor immune response by suppressing immune evasion mechanisms in tumor cells (114, 115).

Together, these findings indicate that MR may have dual functions: suppressing T cell response while spontaneously reducing tumor-mediated suppression of anti-tumor immunity. The net effect of MR on anti-tumor T cell response is therefore likely to be determined by the balance between these two opposing effects. Moreover, the observed synergy between MR and ICB suggests that ICB may shift this balance toward favoring anti-tumor immunity. If so, combining MR and ICB may present a promising strategy in solid tumors.

Conversely, in the absence of ICB, such as in ACT and spontaneous immune response, supplementation with methionine, either intratumorally or systematically, enhances T cell anti-tumor immunity, suggesting that methionine supplementation may improve therapeutic efficacy in these settings (68–70). Methionine supplementation attenuates T cell exhaustion marker expression, including TOX, PD-1 and TIM-3 (70). In the context of chimeric antigen receptor (CAR)-T cell therapy, peritumoral injection of methionine improves efficacy in a syngeneic mouse osteosarcoma model, leading to enhanced tumor control and improved overall survival (70). Consistently, methionine restriction in CAR-T cell-tumor cell cocultures reduces central memory T cell frequency and promotes PD-1 and TIM-3 expression (137). Moreover, dietary methionine restriction impairs human CAR-T cell efficacy against lung squamous cell carcinoma in mice (137). Notably, Bian et al. reported that methionine supplementation enhances T cell response in a small cohort of colorectal cancer patients – oral supplement at 1000 mg/day for two weeks significantly improved CD8+ T cell cytokine production and reduced T cell apoptosis, highlighting the potential for clinical translation (68).

### Manipulating GCN2 to enhance T cell anti-tumor immunity

GCN2 plays a crucial role in regulating T cell immunity, particularly in amino acid-limited conditions and indoleamine 2,3-dioxygenase (IDO)-mediated immune suppression (105, 109, 110, 112). Despite this, relatively few studies have explored the potential of strengthening T cell anti-tumor response through the modulation of GCN2 signaling. A study by St. Paul et al. demonstrated that activation of GCN2 in activated T cells using halo prior to adoptive transfer enhances their anti-tumor activity in murine lymphoma models (113). Importantly, halo treatment induces a similar phenotype in human T cells *in vitro*, suggesting translational potential (113). Consistently, a recent study found that GCN2 activation by dabrafenib, an anticancer reagent, blocks the generation of myeloid-derived suppressor cells (MDSCs) in murine melanoma, indicating that systemic activation of GCN2 may enhance anti-tumor immunity (138). In contrast, another study showed that GCN2 deletion in myeloid cells induces inflammatory macrophages and attenuates MDSC function, leading to enhanced anti-tumor T cell response in murine melanoma models (139). Furthermore, treatment of melanoma-bearing mice with nanoparticles delivering ATF4 siRNA markedly inhibited tumor growth, suggesting that suppression of the GCN2-ATF4 axis may represent a viable therapeutic strategy (139). Together, these conflicting findings underscore the need for future studies to further investigate strategies for modulating GCN2 signaling to strengthen T cell responses in solid tumors.

Temporary conditioning human CAR-T cells in leucine-deprived medium increased the transcription of genes involved in IFN-α response and NF-κB-mediated TNF-α signaling and IFN-γ response. This effect is reversed by GCN2 inhibition, suggesting that GCN2 activation during CAR-T cell manufacturing enhances effector function (140). However, whether this benefit can be translated into improved efficacy against solid tumors remain to be examined.

Clinically, \an GCN2 inhibitor, APL-4098, has been studied in a phase 1 trial for acute myeloid leukemia (AML), where it was well tolerated and reduced AML blast counts in 5 out of 9 of the patients (141). In addition, a phase 1 trial is currently underway to test an GCN2 activator, HC-7366, in AML (142). However, both studies primarily focus on cancer cell–intrinsic effects, it remains unclear how modulating GCN2 signaling influences antitumor T cell responses. Nonetheless, blood samples collected from these clinical trials, if available, would be highly valuable for addressing this question.

**Table 2** provides a summary of the preclinical interventions targeting the GCN2–methionine–ROS pathways.

**Table 2:** Preclinical studies targeting the GCN2–methionine–ROS circuit.

Target axis	Tumor type / model	Intervention strategy	Key outcomes	Ref(s)
GCN2	Melanoma (murine)	GCN2 knockout (T cells)	No impairment of T cell anti-tumor function	(111)
	Glioma (murine)	GCN2 knockout (T cells)	↓ CD8^+^ T cell survival, activation, effector function → ↓ tumor control	(112)
	Lymphoma (murine)	Halofuginone (GCN2 agonist) + ACT	↑ OXPHOS, ↑ effector function, ↑ tumor control	(113)
	Melanoma (murine)	Dabrafenib (GCN2 activation)	↓ MDSCs → enhanced anti-tumor immunity	(138)
	Melanoma (murine)	GCN2 deletion (myeloid) or ATF4 siRNA	↓ MDSCs, ↑ inflammatory macrophages, ↓ tumor growth	(139)
ROS	Melanoma, lymphoma, colon carcinoma, and lung carcinoma	Nrf2 knockout in ACT	↑ tumor killing, ↑ T cell function	(123)
	Colorectal cancer (murine)	Tmed4 knockout	↑ Treg inflammatory phenotype, ↓ FOXP3, ↑ anti-tumor activity	(84)
	Colon cancer & melanoma	Intratumoral H_2_O_2_ + radiotherapy	↓ tumor growth, ↑ IFN-γ, ↑ T cell persistence	(126)
	Viral (LCMV model)	Prdx2 knockout	↑ CD8^+^ T cell effector function and persistence (context-dependent)	(125)
	Lymphoma (ACT)	Glucose restriction (↑ mito ROS)	↑ persistence, complete tumor clearance; ROS-dependent	(8, 127)
	Melanoma (ACT)	NAC treatment	↑ ACT efficacy	(132, 133)
	Blood cancer (murine)	Auranofin	↑ T cell effector function and tumor killing	(124)
	Ovarian cancer (in vitro)	Catalase overexpression (CAR-T)	↑ tumor killing under oxidative stress	(128)
	Breast cancer (in vitro)	TRX1 overexpression (CAR-T)	↑ cytokines, proliferation, cytotoxicity	(31)
	Lymphoma (xenograft)	NRF2 knockdown (CAR-T)	↑ anti-tumor immunity	(124)
Methionine	Colorectal cancer (human/murine)	Methionine supplementation	↑ CD8^+^ T cell function, ↓ apoptosis	(68)
	Prostate cancer (murine) Renal cancer (murine)	Methionine restriction + PD-1 blockade	↑ CD8^+^ T cells, ↑ GZMB/IFNγ, ↓ M2 macrophages	(114)
	Colon cancer (murine)	MR + PD-1 blockade	↑ T cell infiltration, ↑ effector function and cytotoxicity	(115)
	Colorectal cancer (murine)	MR + PD-1 blockade	↑ tumor control	(116)
	Lymphoma (murine)	Transient MR and sustained MR + ACT	↑ IFN-γ, ↑ cytotoxicity (in vitro only) ↓ tumor control and survival (in vivo)	(74)
	Osteosarcoma (murine)	Methionine supplementation + ACT	↑ ACT efficacy	(70)
	Melanoma (murine)	Intermittent MR + ICB	↑ ferroptosis, ↑ T cell infiltration/cytotoxicity	(120)

↓ = decreased; → = leads to; ↑ = increased.

## Section 3: perspectives

It is clear from the existing literature that individually modulating methionine, GCN2, or reactive oxygen species (ROS) does not yield predictable outcomes in the context of T cell immunotherapies. This likely reflects a combination of factors. First, the tumor microenvironment (TME) differs substantially across tumor types [e.g. lymphoma versus pancreatic ductal adenocarcinoma (PDAC)], exerting distinct pressures on T cell metabolism and function and precluding a blanket therapeutic approach (143). For example, a therapeutic approach of activating the GCN2 pathway and increasing antioxidant capacity of T cells may be prudent in the PDAC environment, where oxidative and metabolic stress is high, but may not work for other tumor microenvironments that don’t share the same stress, such as lymphoma and melanoma. Second, the type of T cell–based therapy employed [e.g. immune checkpoint blockade (ICB) versus adoptive T cell therapy (ACT)] is likely to influence how these pathways are engaged. Finally, whether modulation is transient or permanent may play a dominant role in determining therapeutic value. For example, inducing a naïve- or memory-like phenotype during *ex vivo* expansion of ACT products has been shown to be beneficial; however, once transferred into the tumor, these cells must transition to an effector phenotype to mediate tumor killing (144). Here, we will (a) examine approaches to elucidate the GCN2–methionine–ROS circuit to determine what forms of therapeutic modulation for T cell immunotherapies may be beneficial in a given context, and (b) explore different nodes within the GCN2-methionine-ROS circuit where intervention may be advantageous.

### Evaluating the impact of GCN2–methionine–ROS circuit modulation

Given the contradictory literature surrounding interventions in GCN2, methionine, and ROS, it is critical to determine whether targeting these pathways will ultimately enhance T cell function. There are many approaches to address this question. In this review, we focus on multi-omics strategies, CRISPR–Cas9 screening, and drug screening. To extract meaningful insight from these tools, it is essential to develop experimental models that accurately capture relevant environmental contexts. Specifically, the therapeutic setting should closely mirror the therapy of interest [e.g. tumor infiltrating lymphocyte (TIL) therapy, chimeric antigen receptor (CAR)-T cell therapy, PD-1 or CTLA-4 checkpoint blockade]. Likewise, the tumor model, and critically, the TME, must reflect the cancer under study, particularly ones that are less immunogenic or hard to treat. This can be achieved using appropriate syngeneic or xenograft mouse models, as well as *in vitro* systems incorporating patient-derived samples or organoids. Importantly, metabolic interventions are often beneficial in a time-dependent manner (e.g. pre– versus post–adoptive transfer), and this temporal dimension should be explicitly considered when evaluating therapeutic strategies.

Transcriptomics, proteomics, and metabolomics have evolved substantially over the past decades, providing increasingly comprehensive readouts of T cell states. Transcriptomics offers a high-level overview of gene expression and is useful for broadly characterizing cellular phenotypes, such as exhaustion (145). However, it does not capture cellular activity at a functional level. Metabolomics, and to a lesser extent proteomics, can provide a more direct and integrated picture of cellular phenotype (146, 147). Metabolites act as key cellular messengers, conveying signals that tune T cell states and functions (148, 149). As such, profiling cellular metabolite composition can be particularly powerful for understanding T cell phenotypes within specific environments. Together, these omics approaches enable assessment of which pathways are activated or suppressed in T cells under defined conditions, providing insight into whether targeted modulation of a given pathway may be beneficial.

Genome-wide screening offers a complementary and highly scalable approach to interrogate cellular pathways in a systematic manner (150, 151). While CRISPR-Cas9 screens are commonly used to probe a broad range of biological processes, smaller, pathway-focused libraries are increasingly accessible and can be designed to specifically target pathways such as the methionine cycle or GCN2 signaling (152, 153). Using this approach, individual components of a pathway can be perturbed, and the functional consequences of each edit assessed within the same experimental framework. Because CRISPR–Cas9 knockout screens result in permanent gene deletion, this strategy is particularly well suited for evaluating the effects of sustained pathway modulation.

Drug screening provides another powerful, high-throughput method to assess whether modulation of specific genes or pathways enhances T cell function (154, 155). In contrast to CRISPR-based approaches, pharmacological interventions can be inherently transient. This distinction may be especially relevant in ACT settings, where T cells are expanded *ex vivo* prior to infusion and where transient induction of a stem-like or memory-like phenotype has been shown to improve therapeutic outcomes. Drug screens may also be applied after initial tumor–T cell interactions (e.g. in serial killing assays) to identify interventions capable of reinvigorating exhausted T cells.

Furthermore, leveraging clinically available biomarkers may help determine whether modulation of the GCN2–methionine–ROS circuit is beneficial in a given context. For example, several peripheral biomarkers can indirectly reflect ROS levels, such as malondialdehyde (MDA) (156). Similarly, circulating methionine levels can be measured and may serve as an indicator of whether methionine restriction or supplementation would be advantageous within a specific cancer treatment regimen (157).

Engineering the GCN2–methionine–ROS circuit is likely to produce T cell products with context-dependent and potentially unpredictable outcomes. As such, it is essential to employ screening strategies within appropriately defined experimental settings that can capture this complexity.

### Tuning the GCN2–methionine–ROS circuit

There are multiple intervention points within the GCN2–methionine–ROS circuit that may prove useful for enhancing T cell–based immunotherapies, including therapies that act on endogenous T cells (e.g. ICB), against solid tumors. Here, we outline three multiplex strategies that warrant further investigation.

**Figure 3:**
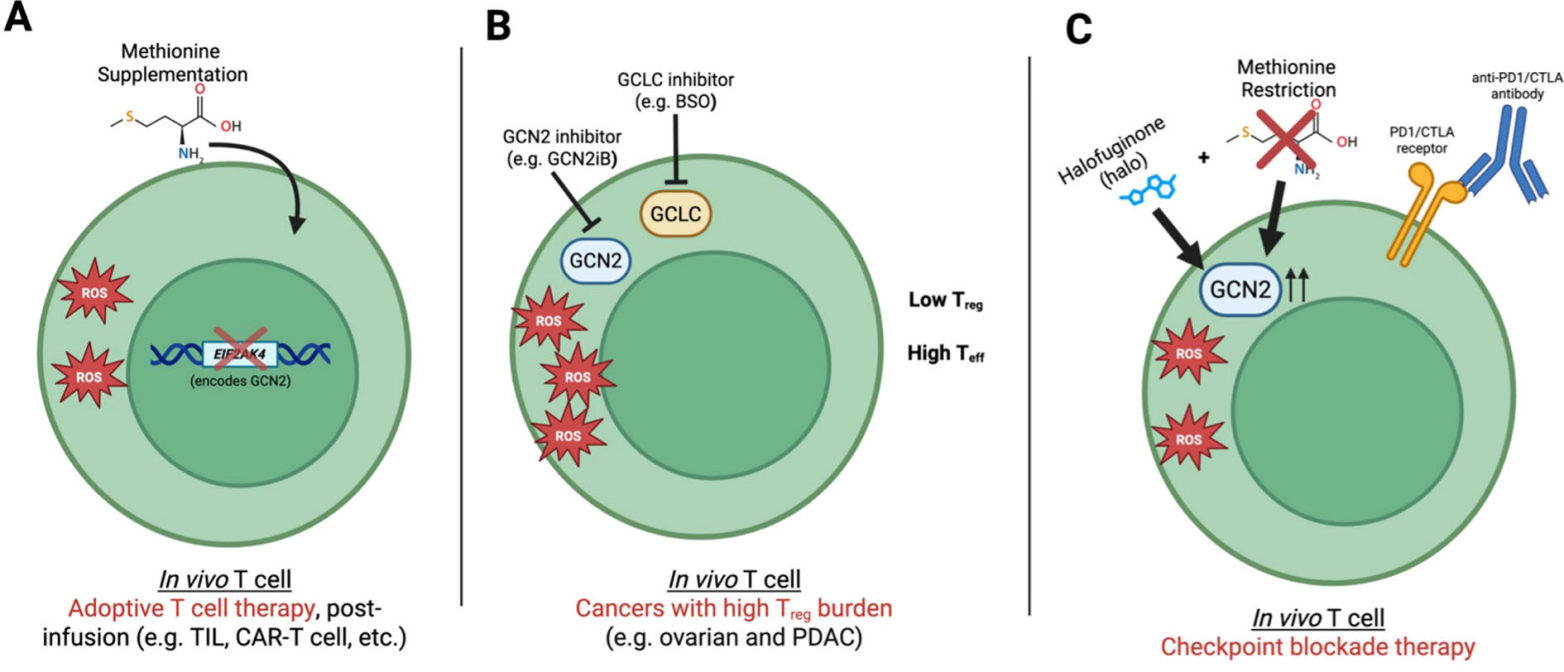
Context-dependent therapeutic tuning of the GCN2–methionine–ROS circuit in T cell–based immunotherapy. **(A)** In adoptive T cell therapy (ACT) paradigms, methionine supplementation combined with genetic deletion of GCN2 suppresses stress-response signaling while sustaining anabolic and mitochondrial activity, thereby tuning intracellular ROS to levels permissive for TCR signaling, effector differentiation, and persistence of transferred T cells (e.g., TILs, CAR-T cells) after infusion. **(B)** In tumors with high regulatory T cell (Treg) burden, combined inhibition of GCN2 and glutathione synthesis (via GCLC inhibition) increases intracellular oxidative stress, destabilizing Treg identity and favoring effector T cell responses. **(C)** In immune checkpoint blockade (ICB) settings, dietary methionine restriction combined with pharmacological activation of GCN2 (e.g., by treating with halofuginone) enhances amino acid stress signaling and antioxidant capacity, potentially synergizing with PD-1/CTLA-4 blockade to support reinvigoration of endogenous T cells in the tumor microenvironment. Together, these models illustrate how therapeutic manipulation of the GCN2–methionine–ROS circuit must be tailored to tumor context, immune composition, and treatment modality.

Within ACT paradigms where T cells are expanded *ex vivo* prior to reinfusion, evidence suggests that methionine restriction can impair T cell function, whereas methionine supplementation may be beneficial (70, 74). Similarly, a study has demonstrated that inhibiting the GCN2 stress response in T cells can enhance adoptively transferred T cell proliferation (109). Mechanistically, these interventions are also likely to reshape intracellular redox balance. Methionine supplementation enhances antioxidant capacity through glutathione biosynthesis and methionine-dependent redox buffering, thereby limiting excessive oxidative stress during rapid *ex vivo* proliferation. Concurrent inhibition or deletion of GCN2 may permit sustained mitochondrial and anabolic activity, which can increase ROS generation, while preventing activation of stress-response pathways that otherwise restrain T cell proliferation and effector differentiation. However, it is worth noting that sustained GCN2 inhibition (e.g. by gene deletion) could potentially lead to T cell exhaustion. Together, these manipulations are predicted to maintain ROS at levels that are permissive for TCR signaling, metabolic activation, and effector programming, without triggering oxidative dysfunction. Thus, combining GCN2 inhibition or deletion with methionine supplementation may improve T cell–based therapies for solid tumors in the ACT setting (**Figure 3A**).

Regulatory T cells (Tregs) have been shown in multiple models to rely on a high antioxidant capacity, characterized by low ROS levels and elevated glutathione (GSH), as well as active GCN2 signaling (158, 159). In cancers where poor prognosis is associated with high endogenous Treg infiltration, a multiplex intervention combining inhibition of both GCN2 (e.g. clinical-grade inhibitors APL-4098 and HC-7366) and glutamate–cysteine ligase catalytic subunit (GCLC) [e.g. clinical-grade inhibitor buthionine sulfoximine (BSO)], the enzyme that synthesizes GSH, may synergistically destabilize Treg phenotypes and promote a more inflammatory state (**Figure 3B**) (160, 161). Mechanistically, this combination is also expected to reduce intracellular methionine availability by increasing anabolic demand (via GCN2 inhibition) while imposing oxidative and metabolic stress (via GCLC inhibition), creating a state of methionine limitation without the normal GCN2-mediated adaptive response.

In the context of ICB, therapeutic efficacy relies on reinvigorating endogenous T cells by blocking inhibitory receptors at the cell surface. Multiple studies have shown that dietary MR can be beneficial in this setting (114, 115). Mechanistically, MR would be expected to indirectly activate GCN2. In this context, combining MR and ICB with an additional intervention, such as a pharmacological GCN2 activator (e.g. halo), may synergistically enhance T cell reinvigoration (**Figure 3C**). MR would limit GSH synthesis, whereas GCN2 signaling would enhance GSH production, potentially maintaining ROS at an optimal, intermediate level. This multiplex strategy may be particularly valuable for solid tumors that have historically been resistant to immunotherapy.

Although tuning the GCN2–methionine–ROS circuit is not straightforward, it represents a potentially powerful strategy when applied in the appropriate context. Moreover, targeting this circuit from multiple angles using multiplex approaches may ultimately prove more effective than single-pathway interventions.

## Conclusions

This review highlights the GCN2–methionine–reactive oxygen species (ROS) axis as an integrated redox-metabolic circuit that plays a central and context-dependent role in shaping T cell anti-tumor immunity. Rather than acting in isolation, GCN2 signaling, methionine metabolism, and reactive oxygen species dynamically intersect to regulate T cell activation, differentiation, metabolic state, and effector function, particularly within the nutrient-poor and oxidative tumor microenvironment. Physiological levels of ROS are essential for sustaining T cell receptor (TCR) signaling, glycolytic reprogramming, and cytotoxic activity, while excessive or prolonged oxidative stress drives exhaustion and apoptosis; GCN2 functions as a key sensor that links amino acid availability and redox stress to adaptive transcriptional and metabolic programs, in part by sustaining glutathione (GSH) synthesis through methionine and cysteine metabolism. Importantly, therapeutic manipulation of any single node in this circuit yields highly variable outcomes that depend on tumor type, microenvironmental pressures, therapy modality (ICB versus ACT), and timing of intervention. As such, blanket metabolic interventions need to be approached with caution and instead emphasizes the need for context-aware, temporally controlled, and multiplex strategies to tune the GCN2–methionine–ROS circuit. Leveraging integrated omics, CRISPR-based perturbation screens, and drug discovery platforms in physiologically relevant models will be essential for identifying when and how modulation of this circuit can be harnessed to enhance T cell–based immunotherapies for solid tumors.

## References

[1] Rangel Rivera GO Knochelmann HM Dwyer CJ Smith AS Wyatt MM Rivera-Reyes AM . Fundamentals of t cell metabolism and strategies to enhance cancer immunotherapy. Front Immunol. (2021) 12:645242. doi: 10.3389/fimmu.2021.645242. PMID: 33815400 PMC8014042

[2] Shi Y Zhang H Miao C . Metabolic reprogram and t cell differentiation in inflammation: current evidence and future perspectives. Cell Death Discov. (2025) 11:123. doi: 10.1038/s41420-025-02403-1. PMID: 40155378 PMC11953409

[3] Jaccard A Wyss T Maldonado-Pérez N Rath JA Bevilacqua A Peng J-J . Reductive carboxylation epigenetically instructs t cell differentiation. Nature. (2023) 621:849–56. doi: 10.1038/s41586-023-06546-y. PMID: 37730993

[4] Li F Liu H Zhang D Ma Y Zhu B . Metabolic plasticity and regulation of t cell exhaustion. Immunology. (2022) 167:482–94. doi: 10.1111/imm.13575. PMID: 36088582

[5] Fultang L Booth S Yogev O Martins da Costa B Tubb V Panetti S . Metabolic engineering against the arginine microenvironment enhances CAR-T cell proliferation and therapeutic activity. Blood. (2020) 136:1155–60. doi: 10.1182/blood.2019004500. PMID: 32573723 PMC7565134

[6] Vigano S Alatzoglou D Irving M Ménétrier-Caux C Caux C Romero P . Targeting adenosine in cancer immunotherapy to enhance t-cell function. Front Immunol. (2019) 10:925. doi: 10.3389/fimmu.2019.00925. PMID: 31244820 PMC6562565

[7] McPhedran SJ Carleton GA Lum JJ . Metabolic engineering for optimized CAR-T cell therapy. Nat Metab. (2024) 6:396–408. doi: 10.1038/s42255-024-00976-2. PMID: 38388705

[8] Klein Geltink RI Edwards-Hicks J Apostolova P O’Sullivan D Sanin DE Patterson AE . Metabolic conditioning of CD8+ effector t cells for adoptive cell therapy. Nat Metab. (2020) 2:703–16. doi: 10.1038/s42255-020-0256-z. PMID: 32747793 PMC10863625

[9] Sukumar M Liu J Ji Y Subramanian M Crompton JG Yu Z . Inhibiting glycolytic metabolism enhances CD8^+^ t cell memory and antitumor function. J Clin Invest. (2013) 123:4479–88. doi: 10.1172/JCI69589. PMID: 24091329 PMC3784544

[10] MacPherson S Keyes S Kilgour MK Smazynski J Chan V Sudderth J . Clinically relevant t cell expansion media activate distinct metabolic programs uncoupled from cellular function. Mol Ther - Methods Clin Dev. (2022) 24:380–93. doi: 10.1016/j.omtm.2022.02.004. PMID: 35284590 PMC8897702

[11] Lennicke C Cochemé HM . Redox metabolism: ROS as specific molecular regulators of cell signaling and function. Mol Cell. (2021) 81:3691–707. doi: 10.1016/j.molcel.2021.08.018. PMID: 34547234

[12] Marchi S Giorgi C Suski JM Agnoletto C Bononi A Bonora M . Mitochondria-ros crosstalk in the control of cell death and aging. J Signal Transduct. (2012) 2012:329635. doi: 10.1155/2012/329635. PMID: 22175013 PMC3235816

[13] Sena LA Li S Jairaman A Prakriya M Ezponda T Hildeman DA . Mitochondria are required for antigen-specific t cell activation through reactive oxygen species signaling. Immunity. (2013) 38:225–36. doi: 10.1016/j.immuni.2012.10.020. PMID: 23415911 PMC3582741

[14] Chen J Liu C Chernatynskaya AV Newby B Brusko TM Xu Y . NADPH oxidase 2–derived reactive oxygen species promote CD8+ t cell effector function. J Immunol. (2024) 212:258–70. doi: 10.4049/jimmunol.2200691. PMID: 38079221 PMC10752859

[15] Bode K Hauri-Hohl M Jaquet V Weyd H . Unlocking the power of NOX2: a comprehensive review on its role in immune regulation. Redox Biol. (2023) 64:102795. doi: 10.1016/j.redox.2023.102795. PMID: 37379662 PMC10320620

[16] Zhaoyun L Wang H Yang C Zhao X Hui L Song J . Enhancing antitumor immunity via ROS-ERS and pyroptosis-induced immunogenic cell death in multiple myeloma. J Immunother Cancer. (2025) 13:e011717. doi: 10.1136/jitc-2025-011717. PMID: 40473272 PMC12142136

[17] Zito E . ERO1: a protein disulfide oxidase and H2O2 producer. Free Radical Biol Med. (2015) 83:299–304. doi: 10.1016/j.freeradbiomed.2015.01.011. PMID: 25651816

[18] Görlach A Bertram K Hudecova S Krizanova O . Calcium and ROS: a mutual interplay. Redox Biol. (2015) 6:260–71. doi: 10.1016/j.redox.2015.08.010. PMID: 26296072 PMC4556774

[19] Zhang Z Zhang L Zhou L Lei Y Zhang Y Huang C . Redox signaling and unfolded protein response coordinate cell fate decisions under ER stress. Redox Biol. (2019) 25:101047. doi: 10.1016/j.redox.2018.11.005. PMID: 30470534 PMC6859529

[20] Bhattarai KR Riaz TA Kim H-R Chae H-J . The aftermath of the interplay between the endoplasmic reticulum stress response and redox signaling. Exp Mol Med. (2021) 53:151–67. doi: 10.1038/s12276-021-00560-8. PMID: 33558590 PMC8080639

[21] Chen S Fan J Xie P Ahn J Fernandez M Billingham LK . CD8^+^ t cells sustain antitumor response by mediating crosstalk between adenosine A2A receptor and glutathione/GPX4. J Clin Invest. (2024) 134:e170071. doi: 10.1172/JCI170071. PMID: 38441967 PMC11014673

[22] Wu G Lupton JR Turner ND Fang Y-Z Yang S . Glutathione metabolism and its implications for health. J Nutr. (2004) 134:489–92. doi: 10.1093/jn/134.3.489. PMID: 14988435

[23] Murphy MP Bayir H Belousov V Chang CJ Davies KJA Davies MJ . Guidelines for measuring reactive oxygen species and oxidative damage in cells and *in vivo*. Nat Metab. (2022) 4:651–62. doi: 10.1038/s42255-022-00591-z. PMID: 35760871 PMC9711940

[24] Mak TW Grusdat M Duncan GS Dostert C Nonnenmacher Y Cox M . Glutathione primes t cell metabolism for inflammation. Immunity. (2017) 46:675–89. doi: 10.1016/j.immuni.2017.03.019. PMID: 28423341

[25] Averill-Bates DA . The antioxidant glutathione. In: Vitamins and Hormones. San Diego, CA: Academic Press. p. 109–41. doi: 10.1016/bs.vh.2022.09.002 36707132

[26] Franco R Cidlowski JA . Apoptosis and glutathione: beyond an antioxidant. Cell Death Differ. (2009) 16:1303–14. doi: 10.1038/cdd.2009.107. PMID: 19662025

[27] Stöcker S Maurer M Ruppert T Dick TP . A role for 2-Cys peroxiredoxins in facilitating cytosolic protein thiol oxidation. Nat Chem Biol. (2018) 14:148–55. doi: 10.1038/nchembio.2536. PMID: 29251718 PMC5863949

[28] Perkins A Nelson KJ Parsonage D Poole LB Karplus PA . Peroxiredoxins: guardians against oxidative stress and modulators of peroxide signaling. Trends Biochem Sci. (2015) 40:435–45. doi: 10.1016/j.tibs.2015.05.001. PMID: 26067716 PMC4509974

[29] Bolduc J Koruza K Luo T Malo Pueyo J Vo TN Ezeriņa D . Peroxiredoxins wear many hats: factors that fashion their peroxide sensing personalities. Redox Biol. (2021) 42:101959. doi: 10.1016/j.redox.2021.101959. PMID: 33895094 PMC8113037

[30] Lee W Choi K-S Riddell J Ip C Ghosh D Park J-H . Human peroxiredoxin 1 and 2 are not duplicate proteins: the unique presence of CYS83 in Prx1 underscores the structural and functional differences between Prx1 and Prx2 *. J Biol Chem. (2007) 282:22011–22. doi: 10.1074/jbc.M610330200. PMID: 17519234

[31] Chakraborty P Chatterjee S Kesarwani P Thyagarajan K Iamsawat S Dalheim A . Thioredoxin-1 improves the immunometabolic phenotype of antitumor t cells. J Biol Chem. (2019) 294:9198–212. doi: 10.1074/jbc.RA118.006753. PMID: 30971427 PMC6556575

[32] Case AJ McGill JL Tygrett LT Shirasawa T Spitz DR Waldschmidt TJ . Elevated mitochondrial superoxide disrupts normal t-cell development to impair adaptive immune responses to an influenza challenge. Free Radic Biol Med. (2011) 50:448–58. doi: 10.1016/j.freeradbiomed.2010.11.025. PMID: 21130157 PMC3026081

[33] Loopmans S Rohlenova K van Brussel T Stockmans I Moermans K Peredo N . The pentose phosphate pathway controls oxidative protein folding and prevents ferroptosis in chondrocytes. Nat Metab. (2025) 7:182–95. doi: 10.1038/s42255-024-01187-5. PMID: 39794539 PMC11774761

[34] Dahabieh MS DeCamp LM Oswald BM Kitchen-Goosen SM Fu Z Vos M . The prostacyclin receptor PTGIR is a NRF2-dependent regulator of CD8+ t cell exhaustion. Nat Immunol. (2025) 26:1139–51. doi: 10.1038/s41590-025-02185-9. PMID: 40579556 PMC12208871

[35] Lin W Shen P Song Y Huang Y Tu S . Reactive oxygen species in autoimmune cells: function, differentiation, and metabolism. Front Immunol. (2021) 12:635021. doi: 10.3389/fimmu.2021.635021. PMID: 33717180 PMC7946999

[36] Venza I Venza M Visalli M Lentini G Teti D d’Alcontres FS . ROS as regulators of cellular processes in melanoma. Oxid Med Cell Longev. (2021) 2021:1208690. doi: 10.1155/2021/1208690. PMID: 34725562 PMC8557056

[37] Weinberg F Ramnath N Nagrath D . Reactive oxygen species in the tumor microenvironment: an overview. Cancers (Basel). (2019) 11:1191. doi: 10.3390/cancers11081191. PMID: 31426364 PMC6721577

[38] Abdel Hadi N Reyes-Castellanos G Carrier A . Targeting redox metabolism in pancreatic cancer. Int J Mol Sci. (2021) 22:1534. doi: 10.3390/ijms22041534. PMID: 33546421 PMC7913542

[39] ArulJothi KN Kumaran K Senthil S Nidhu AB Munaff N Janitri VB . Implications of reactive oxygen species in lung cancer and exploiting it for therapeutic interventions. Med Oncol. (2023) 40:43. doi: 10.1007/s12032-022-01900-y. PMID: 36472716 PMC9734980

[40] Weinberg SE Chandel NS . Mitochondria reactive oxygen species signaling in immune responses. Immunity. (2025) 58:1904–21. doi: 10.1016/j.immuni.2025.07.012. PMID: 40763730 PMC12371701

[41] Kesarwani P Murali AK Al-Khami AA Mehrotra S . Redox regulation of t-cell function: from molecular mechanisms to significance in human health and disease. Antioxid Redox Signal. (2013) 18:1497–534. doi: 10.1089/ars.2011.4073. PMID: 22938635 PMC3603502

[42] Kong H Chandel NS . Regulation of redox balance in cancer and t cells. J Biol Chem. (2018) 293:7499–507. doi: 10.1074/jbc.TM117.000257. PMID: 29282291 PMC5961053

[43] Piecyk M Ferraro-Peyret C Laville D Perros F Chaveroux C . Novel insights into the GCN2 pathway and its targeting. therapeutic value in cancer and lessons from lung fibrosis development. FEBS J. (2024) 291:4867–89. doi: 10.1111/febs.17203. PMID: 38879870

[44] Romano PR Garcia-Barrio MT Zhang X Wang Q Taylor DR Zhang F . Autophosphorylation in the activation loop is required for full kinase activity *in vivo* of human and yeast eukaryotic initiation factor 2alpha kinases PKR and GCN2. Mol Cell Biol. (1998) 18:2282–97. doi: 10.1128/MCB.18.4.2282. PMID: 9528799 PMC121479

[45] Dever TE Feng L Wek RC Cigan AM Donahue TF Hinnebusch AG . Phosphorylation of initiation factor 2 alpha by protein kinase GCN2 mediates gene-specific translational control of GCN4 in yeast. Cell. (1992) 68:585–96. doi: 10.1016/0092-8674(92)90193-g. PMID: 1739968

[46] Scorsone KA Panniers R Rowlands AG Henshaw EC . Phosphorylation of eukaryotic initiation factor 2 during physiological stresses which affect protein synthesis. J Biol Chem. (1987) 262:14538–43. doi: 10.1016/s0021-9258(18)47829-5 3667588

[47] Sood R Porter AC Olsen DA Cavener DR Wek RC . A mammalian homologue of GCN2 protein kinase important for translational control by phosphorylation of eukaryotic initiation factor-2alpha. Genetics. (2000) 154:787–801. doi: 10.1093/genetics/154.2.787. PMID: 10655230 PMC1460965

[48] Neill G Masson GR . A stay of execution: ATF4 regulation and potential outcomes for the integrated stress response. Front Mol Neurosci. (2023) 16:1112253. doi: 10.3389/fnmol.2023.1112253. PMID: 36825279 PMC9941348

[49] Vattem KM Wek RC . Reinitiation involving upstream ORFs regulates ATF4 mRNA translation in mammalian cells. Proc Natl Acad Sci USA. (2004) 101:11269–74. doi: 10.1073/pnas.0400541101. PMID: 15277680 PMC509193

[50] Harding HP Zhang Y Zeng H Novoa I Lu PD Calfon M . An integrated stress response regulates amino acid metabolism and resistance to oxidative stress. Mol Cell. (2003) 11:619–33. doi: 10.1016/s1097-2765(03)00105-9. PMID: 12667446

[51] Ye J Kumanova M Hart LS Sloane K Zhang H De Panis DN . The GCN2-ATF4 pathway is critical for tumour cell survival and proliferation in response to nutrient deprivation. EMBO J. (2010) 29:2082–96. doi: 10.1038/emboj.2010.81. PMID: 20473272 PMC2892366

[52] Tang CP Clark O Ferrarone JR Campos C Lalani AS Chodera JD . GCN2 kinase activation by ATP-competitive kinase inhibitors. Nat Chem Biol. (2022) 18:207–15. doi: 10.1038/s41589-021-00947-8. PMID: 34949839 PMC9549920

[53] Jin H-O Hong S-E Kim J-Y Jang S-K Park I-C . Amino acid deprivation induces AKT activation by inducing GCN2/ATF4/REDD1 axis. Cell Death Dis. (2021) 12:1127. doi: 10.1038/s41419-021-04417-w. PMID: 34862383 PMC8642548

[54] Ye J Palm W Peng M King B Lindsten T Li MO . GCN2 sustains mTORC1 suppression upon amino acid deprivation by inducing Sestrin2. Genes Dev. (2015) 29:2331–6. doi: 10.1101/gad.269324.115. PMID: 26543160 PMC4691887

[55] Maurin A-C Parry L B’chir W Carraro V Coudy-Gandilhon C Chaouki G . GCN2 upregulates autophagy in response to short-term deprivation of a single essential amino acid. Autophagy Rep. (2022) 1:119–42. doi: 10.1080/27694127.2022.2049045. PMID: 40396040 PMC11864613

[56] B’chir W Maurin A-C Carraro V Averous J Jousse C Muranishi Y . The eIF2α/ATF4 pathway is essential for stress-induced autophagy gene expression. Nucleic Acids Res. (2013) 41:7683–99. doi: 10.1093/nar/gkt563. PMID: 23804767 PMC3763548

[57] Zhu H-L Shi X-T Xu X-F Zhou G-X Xiong Y-W Yi S-J . Melatonin protects against environmental stress-induced fetal growth restriction via suppressing ROS-mediated GCN2/ATF4/BNIP3-dependent mitophagy in placental trophoblasts. Redox Biol. (2021) 40:101854. doi: 10.1016/j.redox.2021.101854. PMID: 33454563 PMC7811044

[58] Chourasia AH Tracy K Frankenberger C Boland ML Sharifi MN Drake LE . Mitophagy defects arising from BNip3 loss promote mammary tumor progression to metastasis. EMBO Rep. (2015) 16:1145–63. doi: 10.15252/embr.201540759. PMID: 26232272 PMC4576983

[59] Chaveroux C Lambert-Langlais S Parry L Carraro V Jousse C Maurin A-C . Identification of GCN2 as new redox regulator for oxidative stress prevention *in vivo*. Biochem Biophys Res Commun. (2011) 415:120–4. doi: 10.1016/j.bbrc.2011.10.027. PMID: 22020073

[60] Toboz P Amiri M Tabatabaei N Dufour CR Kim SH Fillebeen C . The amino acid sensor GCN2 controls red blood cell clearance and iron metabolism through regulation of liver macrophages. Proc Natl Acad Sci. (2022) 119:e2121251119. doi: 10.1073/pnas.2121251119. PMID: 35994670 PMC9436309

[61] Zhu J Berisa M Schwörer S Qin W Cross JR Thompson CB . Transsulfuration activity can support cell growth upon extracellular cysteine limitation. Cell Metab. (2019) 30:865–876.e5. doi: 10.1016/j.cmet.2019.09.009. PMID: 31607565 PMC6961654

[62] Wang ST Chen HW Sheen LY Lii CK . Methionine and cysteine affect glutathione level, glutathione-related enzyme activities and the expression of glutathione S-transferase isozymes in rat hepatocytes. J Nutr. (1997) 127:2135–41. doi: 10.1093/jn/127.11.2135. PMID: 9372907

[63] Wang W Zou W . Amino acids and their transporters in T cell immunity and cancer therapy. Mol Cell. (2020) 80:384–95. doi: 10.1016/j.molcel.2020.09.006. PMID: 32997964 PMC7655528

[64] Garg SK Yan Z Vitvitsky V Banerjee R . Differential dependence on cysteine from transsulfuration versus transport during T cell activation. Antioxid Redox Signal. (2011) 15:39–47. doi: 10.1089/ars.2010.3496. PMID: 20673163 PMC3110100

[65] Zhao T Lum JJ . Methionine cycle-dependent regulation of T cells in cancer immunity. Front Oncol. (2022) 12:969563. doi: 10.3389/fonc.2022.969563. PMID: 36033438 PMC9399763

[66] Sinclair LV Howden AJ Brenes A Spinelli L Hukelmann JL Macintyre AN . Antigen receptor control of methionine metabolism in T cells. Elife. (2019) 8:e44210. doi: 10.7554/eLife.44210. PMID: 30916644 PMC6497464

[67] Roy DG Chen J Mamane V Ma EH Muhire BM Sheldon RD . Methionine metabolism shapes T helper cell responses through regulation of epigenetic reprogramming. Cell Metab. (2020) 31:250–266.e9. doi: 10.1016/j.cmet.2020.01.006. PMID: 32023446

[68] Bian Y Li W Kremer DM Sajjakulnukit P Li S Crespo J . Cancer SLC43A2 alters T cell methionine metabolism and histone methylation. Nature. (2020) 585:277–82. doi: 10.1038/s41586-020-2682-1. PMID: 32879489 PMC7486248

[69] Pandit M Kil Y-S Ahn J-H Pokhrel RH Gu Y Mishra S . Methionine consumption by cancer cells drives a progressive upregulation of PD-1 expression in CD4 T cells. Nat Commun. (2023) 14:2593. doi: 10.1038/s41467-023-38316-9. PMID: 37147330 PMC10162977

[70] Sharma P Guo A Poudel S Boada-Romero E Verbist KC Palacios G . Early methionine availability attenuates T cell exhaustion. Nat Immunol. (2025) 26:1384–96. doi: 10.1038/s41590-025-02223-6. PMID: 40702340 PMC12307228

[71] Puleston DJ Baixauli F Sanin DE Edwards-Hicks J Villa M Kabat AM . Polyamine metabolism is a central determinant of helper T cell lineage fidelity. Cell. (2021) 184:4186–4202.e20. doi: 10.1016/j.cell.2021.06.007. PMID: 34216540 PMC8358979

[72] Hayes CS Shicora AC Keough MP Snook AE Burns MR Gilmour SK . Polyamine-blocking therapy reverses immunosuppression in the tumor microenvironment. Cancer Immunol Res. (2014) 2:274–85. doi: 10.1158/2326-6066.CIR-13-0120-T. PMID: 24778323 PMC4101915

[73] Hibino S Eto S Hangai S Endo K Ashitani S Sugaya M . Tumor cell-derived spermidine is an oncometabolite that suppresses TCR clustering for intratumoral CD8+ T cell activation. Proc Natl Acad Sci USA. (2023) 120:e2305245120. doi: 10.1073/pnas.2305245120. PMID: 37276392 PMC10268234

[74] Zhao T Carleton GA Macpherson S Shiyuk M Monaghan J Han J . Methionine regulates antitumor function of CD8+ T cells through polyamine synthesis. (2025). doi: 10.1101/2025.10.03.680201 PMC13636119 42503479

[75] Buckler JL Liu X Turka LA . Regulation of T-cell responses by PTEN. Immunol Rev. (2008) 224:239–48. doi: 10.1111/j.1600-065X.2008.00650.x. PMID: 18759931 PMC2876726

[76] Trinh VH Nguyen Huu T Sah DK Choi JM Yoon HJ Park SC . Redox regulation of PTEN by reactive oxygen species: its role in physiological processes. Antioxidants (Basel). (2024) 13:199. doi: 10.3390/antiox13020199. PMID: 38397797 PMC10886030

[77] Ben‐Kasus Nissim T Zhang X Elazar A Roy S Stolwijk JA Zhou Y . Mitochondria control store‐operated Ca2+ entry through Na+ and redox signals. EMBO J. (2017) 36:797–815. doi: 10.15252/embj.201592481. PMID: 28219928 PMC5350565

[78] Hogan PG . Calcium–NFAT transcriptional signalling in T cell activation and T cell exhaustion. Cell Calcium. (2017) 63:66–9. doi: 10.1016/j.ceca.2017.01.014. PMID: 28153342 PMC5739523

[79] Shu P Liang H Zhang J Lin Y Chen W Zhang D . Reactive oxygen species formation and its effect on CD4+ T cell-mediated inflammation. Front Immunol. (2023) 14:1199233. doi: 10.3389/fimmu.2023.1199233. PMID: 37304262 PMC10249013

[80] Weigelin B Friedl P . T cell-mediated additive cytotoxicity – death by multiple bullets. Trends Cancer. (2022) 8:980–7. doi: 10.1016/j.trecan.2022.07.007. PMID: 35965200

[81] Montauti E Oh DY Fong L . CD4+ T cells in antitumor immunity. Trends Cancer. (2024) 10:969–85. doi: 10.1016/j.trecan.2024.07.009. PMID: 39242276 PMC11464182

[82] Dang EV Barbi J Yang H-Y Jinasena D Yu H Zheng Y . Control of TH17/Treg balance by hypoxia-inducible factor 1. Cell. (2011) 146:772–84. doi: 10.1016/j.cell.2011.07.033. PMID: 21871655 PMC3387678

[83] Obata F Hoshino A Toyama A . Hydrogen peroxide increases interleukin-12 p40/p70 molecular ratio and induces Th2-predominant responses in mice. Scand J Immunol. (2006) 63:125–30. doi: 10.1111/j.1365-3083.2005.01718.x. PMID: 16476011

[84] Jiang Z Wang H Wang X Duo H Tao Y Li J . TMED4 facilitates regulatory T cell suppressive function via ROS homeostasis in tumor and autoimmune mouse models. J Clin Invest. (2025) 21:1247–1263. doi: 10.1172/JCI179874. PMID: 39480507 PMC11684806

[85] Vaikunthanathan T Landmann E Correa DM Romano M Trevelin SC Peng Q . Dysregulated anti-oxidant signalling and compromised mitochondrial integrity negatively influence regulatory T cell function and viability in liver disease. eBioMedicine. (2023) 95:104778. doi: 10.1016/j.ebiom.2023.104778. PMID: 37657135 PMC10480539

[86] Scharping NE Rivadeneira DB Menk AV Vignali PDA Ford BR Rittenhouse NL . Mitochondrial stress induced by continuous stimulation under hypoxia rapidly drives T cell exhaustion. Nat Immunol. (2021) 22:205–15. doi: 10.1038/s41590-020-00834-9. PMID: 33398183 PMC7971090

[87] Vardhana SA Hwee MA Berisa M Wells DK Yost KE King B . Impaired mitochondrial oxidative phosphorylation limits the self-renewal of T cells exposed to persistent antigen. Nat Immunol. (2020) 21:1022–33. doi: 10.1038/s41590-020-0725-2. PMID: 32661364 PMC7442749

[88] Steinert EM Furtado Bruza B Danchine VD Grant RA Vasan K Kharel A . Mitochondrial respiration is necessary for CD8+ T cell proliferation and cell fate. Nat Immunol. (2025) 26:1267–74. doi: 10.1038/s41590-025-02202-x. PMID: 40670617 PMC12307223

[89] Toledano Zur R Atar O Barliya T Hoogi S Abramovich I Gottlieb E . Genetically engineering glycolysis in T cells increases their antitumor function. J Immunother Cancer. (2024) 12:e008434. doi: 10.1136/jitc-2023-008434. PMID: 38964783 PMC11227835

[90] Hu Z Qu G Yu X Jiang H Teng X-L Ding L . Acylglycerol kinase maintains metabolic state and immune responses of CD8+ T cells. Cell Metab. (2019) 30:290–302.e5. doi: 10.1016/j.cmet.2019.05.016. PMID: 31204281

[91] Luan F Li Y Ning J Tran JT Blane TR Bhargava R . Loss of Bcl6 promotes antitumor immunity by activating glycolysis to rescue CD8 T-cell function. Life Sci Alliance. (2026) 9 (1):e202503335. doi: 10.26508/lsa.202503335. PMID: 41145211 PMC12559151

[92] Ushio-Fukai M Alexander RW Akers M Yin Q Fujio Y Walsh K . Reactive oxygen species mediate the activation of Akt/protein kinase B by angiotensin II in vascular smooth muscle cells. J Biol Chem. (1999) 274:22699–704. doi: 10.1074/jbc.274.32.22699. PMID: 10428852

[93] Chen T Xie S Cheng J Zhao Q Wu H Jiang P . AKT1 phosphorylation of cytoplasmic ME2 induces a metabolic switch to glycolysis for tumorigenesis. Nat Commun. (2024) 15:686. doi: 10.1038/s41467-024-44772-8. PMID: 38263319 PMC10805786

[94] Li M Zhao L Liu J Liu A Jia C Ma D . Multi-mechanisms are involved in reactive oxygen species regulation of mTORC1 signaling. Cell Signalling. (2010) 22:1469–76. doi: 10.1016/j.cellsig.2010.05.015. PMID: 20639120

[95] Yalcin S Marinkovic D Mungamuri SK Zhang X Tong W Sellers R . ROS‐mediated amplification of AKT/mTOR signalling pathway leads to myeloproliferative syndrome in Foxo3-/- mice. EMBO J. (2010) 29:4118–31. doi: 10.1038/emboj.2010.292. PMID: 21113129 PMC3018793

[96] Jung S-N Yang WK Kim J Kim HS Kim EJ Yun H . Reactive oxygen species stabilize hypoxia-inducible factor-1 alpha protein and stimulate transcriptional activity via AMP-activated protein kinase in DU145 human prostate cancer cells. Carcinogenesis. (2008) 29:713–21. doi: 10.1093/carcin/bgn032. PMID: 18258605

[97] Kostel Bal S Giuliani S Block J Repiscak P Hafemeister C Shahin T . Biallelic NFATC1 mutations cause an inborn error of immunity with impaired CD8+ T-cell function and perturbed glycolysis. Blood. (2023) 142:827–45. doi: 10.1182/blood.2022018303. PMID: 37249233

[98] Wang M Chen Z Zhang Y . CBP/p300 and HDAC activities regulate H3K27 acetylation dynamics and zygotic genome activation in mouse preimplantation embryos. EMBO J. (2022) 41:e112012. doi: 10.15252/embj.2022112012. PMID: 36215692 PMC9670200

[99] Zhang Z Wang X Hamdan FH Likhobabina A Patil S Aperdannier L . NFATc1 is a central mediator of EGFR-induced ARID1A chromatin dissociation during acinar cell reprogramming. Cell Mol Gastroenterol Hepatol. (2023) 15:1219–46. doi: 10.1016/j.jcmgh.2023.01.015. PMID: 36758798 PMC10064440

[100] Levring TB Hansen AK Nielsen BL Kongsbak M von Essen MR Woetmann A . Activated human CD4+ T cells express transporters for both cysteine and cystine. Sci Rep. (2012) 2:266. doi: 10.1038/srep00266. PMID: 22355778 PMC3278673

[101] Ma EH Bantug G Griss T Condotta S Johnson RM Samborska B . Serine is an essential metabolite for effector T cell expansion. Cell Metab. (2017) 25:345–57. doi: 10.1016/j.cmet.2016.12.011. PMID: 28111214

[102] Ma EH Dahabieh MS DeCamp LM Kaymak I Kitchen-Goosen SM Roy DG . 13C metabolite tracing reveals glutamine and acetate as critical *in vivo* fuels for CD8+ T cells. bioRxiv. (2023), 2023.06.09.544407. doi: 10.1101/2023.06.09.544407. PMID: 38809979 PMC11135420

[103] Geiger R Rieckmann JC Wolf T Basso C Feng Y Fuhrer T . L-arginine modulates T cell metabolism and enhances survival and anti-tumor activity. Cell. (2016) 167:829–842.e13. doi: 10.1016/j.cell.2016.09.031. PMID: 27745970 PMC5075284

[104] Lee GK Park HJ Macleod M Chandler P Munn DH Mellor AL . Tryptophan deprivation sensitizes activated T cells to apoptosis prior to cell division. Immunology. (2002) 107:452–60. doi: 10.1046/j.1365-2567.2002.01526.x. PMID: 12460190 PMC1782830

[105] Fallarino F Grohmann U You S McGrath BC Cavener DR Vacca C . The combined effects of tryptophan starvation and tryptophan catabolites down-regulate T cell receptor zeta-chain and induce a regulatory phenotype in naive T cells. J Immunol. (2006) 176:6752–61. doi: 10.4049/jimmunol.176.11.6752. PMID: 16709834

[106] Kamphorst JJ Nofal M Commisso C Hackett SR Lu W Grabocka E . Human pancreatic cancer tumors are nutrient poor and tumor cells actively scavenge extracellular protein. Cancer Res. (2015) 75:544–53. doi: 10.1158/0008-5472.CAN-14-2211. PMID: 25644265 PMC4316379

[107] Sullivan MR Danai LV Lewis CA Chan SH Gui DY Kunchok T . Quantification of microenvironmental metabolites in murine cancers reveals determinants of tumor nutrient availability. Elife. (2019) 8:e44235. doi: 10.7554/eLife.44235. PMID: 30990168 PMC6510537

[108] Vecchio E Caiazza C Mimmi S Avagliano A Iaccino E Brusco T . Metabolites profiling of melanoma interstitial fluids reveals uridine diphosphate as potent immune modulator capable of limiting tumor growth. Front Cell Dev Biol. (2021) 9:730726. doi: 10.3389/fcell.2021.730726. PMID: 34604232 PMC8486041

[109] Munn DH Sharma MD Baban B Harding HP Zhang Y Ron D . GCN2 kinase in T cells mediates proliferative arrest and anergy induction in response to indoleamine 2,3-dioxygenase. Immunity. (2005) 22:633–42. doi: 10.1016/j.immuni.2005.03.013. PMID: 15894280

[110] Van de Velde L-A Guo X-Z Barbaric L Smith AM Oguin TH Thomas PG . Stress kinase GCN2 controls the proliferative fitness and trafficking of cytotoxic T cells independent of environmental amino acid sensing. Cell Rep. (2016) 17:2247–58. doi: 10.1016/j.celrep.2016.10.079. PMID: 27880901 PMC5131879

[111] Sonner JK Deumelandt K Ott M Thomé CM Rauschenbach KJ Schulz S . The stress kinase GCN2 does not mediate suppression of antitumor T cell responses by tryptophan catabolism in experimental melanomas. Oncoimmunology. (2016) 5:e1240858. doi: 10.1080/2162402X.2016.1240858. PMID: 28123877 PMC5214097

[112] Rashidi A Miska J Lee-Chang C Kanojia D Panek WK Lopez-Rosas A . GCN2 is essential for CD8+ T cell survival and function in murine models of Malignant glioma. Cancer Immunol Immunother. (2020) 69:81–94. doi: 10.1007/s00262-019-02441-6. PMID: 31844909 PMC6952559

[113] St Paul M Saibil SD Kates M Han S Lien SC Laister RC . Ex vivo activation of the GCN2 pathway metabolically reprograms T cells, leading to enhanced adoptive cell therapy. Cell Rep Med. (2024) 5:101465. doi: 10.1016/j.xcrm.2024.101465. PMID: 38460518 PMC10983112

[114] Orillion A Damayanti NP Shen L Adelaiye-Ogala R Affronti H Elbanna M . Dietary protein restriction reprograms tumor-associated macrophages and enhances immunotherapy. Clin Cancer Res. (2018) 24:6383–95. doi: 10.1158/1078-0432.CCR-18-0980. PMID: 30190370 PMC6455918

[115] Li T Tan Y-T Chen Y-X Zheng X-J Wang W Liao K . Methionine deficiency facilitates antitumour immunity by altering m6A methylation of immune checkpoint transcripts. Gut. (2023) 72:501–11. doi: 10.1136/gutjnl-2022-326928. PMID: 35803704 PMC9933173

[116] Wang Q-L Chen Z Lu X Lin H Feng H Weng N . Methionine metabolism dictates PCSK9 expression and antitumor potency of PD-1 blockade in MSS colorectal cancer. Adv Sci. (2025) 12:2501623. doi: 10.1002/advs.202501623. PMID: 40125618 PMC12097065

[117] Fang L Hao Y Yu H Gu X Peng Q Zhuo H . Methionine restriction promotes cGAS activation and chromatin untethering through demethylation to enhance antitumor immunity. Cancer Cell. (2023) 41:1118–1133.e12. doi: 10.1016/j.ccell.2023.05.005. PMID: 37267951

[118] Morehead LC Garg S Wallis KF Siegel ER Tackett AJ Miousse IR . Increased response to immune checkpoint inhibitors with dietary methionine restriction. bioRxiv. (2023), 2023.04.05.535695. doi: 10.1101/2023.04.05.535695. PMID: 37760436 PMC10526448

[119] Ji M Xu X Xu Q Hsiao Y-C Martin C Ukraintseva S . Methionine restriction-induced sulfur deficiency impairs antitumour immunity partially through gut microbiota. Nat Metab. (2023) 5:1526–43. doi: 10.1038/s42255-023-00854-3. PMID: 37537369 PMC10513933

[120] Xue Y Lu F Chang Z Li J Gao Y Zhou J . Intermittent dietary methionine deprivation facilitates tumoral ferroptosis and synergizes with checkpoint blockade. Nat Commun. (2023) 14:4758. doi: 10.1038/s41467-023-40518-0. PMID: 37553341 PMC10409767

[121] Lee BC Gladyshev VN . The biological significance of methionine sulfoxide stereochemistry. Free Radic Biol Med. (2011) 50:221–7. doi: 10.1016/j.freeradbiomed.2010.11.008. PMID: 21075204 PMC3311537

[122] Jo Y Lee B Joo M Hong C . Nrf2 expression is upregulated in tumor infiltrating T cells and induces T cell anergy. J Immunol. (2016) 196:143.15. doi: 10.4049/jimmunol.196.Supp.143.15

[123] Jo Y Shim JA Jeong JW Kim H Lee SM Jeong J . Targeting ROS-sensing Nrf2 potentiates anti-tumor immunity of intratumoral CD8+ T and CAR-T cells. Mol Ther. (2024) 32:3879–94. doi: 10.1016/j.ymthe.2024.08.019. PMID: 39169624 PMC11573615

[124] Renken S Nakajima T Magalhaes I Mattsson J Lundqvist A Arnér ESJ . Targeting of Nrf2 improves antitumoral responses by human NK cells, TIL and CAR T cells during oxidative stress. J Immunother Cancer. (2022) 10:e004458. doi: 10.1136/jitc-2021-004458. PMID: 35738800 PMC9226989

[125] Michalek RD Crump KE Weant AE Hiltbold EM Juneau DG Moon E-Y . Peroxiredoxin II regulates effector and secondary memory CD8+ T cell responses. J Virol. (2012) 86:13629–41. doi: 10.1128/JVI.01559-12. PMID: 23055551 PMC3503052

[126] Kemmotsu N Zhu L Nagasaki J Otani Y Ueda Y Dansako H . Combination therapy with hydrogen peroxide and irradiation promotes an abscopal effect in mouse models. Cancer Sci. (2023) 114:3848–56. doi: 10.1111/cas.15911. PMID: 37485636 PMC10551598

[127] Oh JH Cederberg RA Tanaka E Bopp L Ser T Niyyati S . Inducing an oxidized redox-balance improves anti-tumor CD8+ T cell function. (2023), 2023.03.27.533229. doi: 10.1101/2023.03.27.533229

[128] Ligtenberg MA Mougiakakos D Mukhopadhyay M Witt K Lladser A Chmielewski M . Coexpressed catalase protects chimeric antigen receptor–redirected T cells as well as bystander cells from oxidative stress–induced loss of antitumor activity. J Immunol. (2016) 196:759–66. doi: 10.4049/jimmunol.1401710. PMID: 26673145 PMC4705591

[129] Shah MA Rogoff HA . Implications of reactive oxygen species on cancer formation and its treatment. Semin Oncol. (2021) 48:238–45. doi: 10.1053/j.seminoncol.2021.05.002. PMID: 34548190

[130] Habtetsion T Ding Z-C Pi W Li T Lu C Chen T . Alteration of tumor metabolism by CD4+ T cells leads to TNF-α-dependent intensification of oxidative stress and tumor cell death. Cell Metab. (2018) 28:228–242.e6. doi: 10.1016/j.cmet.2018.05.012. PMID: 29887396 PMC6082691

[131] Fauskanger M Haabeth OAW Skjeldal FM Bogen B Tveita AA . Tumor killing by CD4+ T cells is mediated via induction of inducible nitric oxide synthase-dependent macrophage cytotoxicity. Front Immunol. (2018) 9:1684. doi: 10.3389/fimmu.2018.01684. PMID: 30083157 PMC6064871

[132] Scheffel MJ Scurti G Wyatt MM Garrett-Mayer E Paulos CM Nishimura MI . N-acetyl cysteine protects anti-melanoma cytotoxic T cells from exhaustion induced by rapid expansion via the downmodulation of Foxo1 in an Akt-dependent manner. Cancer Immunol Immunother. (2018) 67:691–702. doi: 10.1007/s00262-018-2120-5. PMID: 29396710 PMC5862784

[133] Scheffel MJ Scurti G Simms P Garrett-Mayer E Mehrotra S Nishimura MI . Efficacy of adoptive T-cell therapy is improved by treatment with the antioxidant N-acetyl cysteine, which limits activation-induced T-cell death. Cancer Res. (2016) 76:6006–16. doi: 10.1158/0008-5472.CAN-16-0587. PMID: 27742673 PMC5074089

[134] Yamashita M . Auranofin: Past to present, and repurposing. Int Immunopharmacol. (2021) 101:108272. doi: 10.1016/j.intimp.2021.108272. PMID: 34731781

[135] Tenório MS Graciliano NG Moura FA de Oliveira ACM Goulart MOF . N-acetylcysteine (NAC): Impacts on human health. Antioxidants (Basel). (2021) 10:967. doi: 10.3390/antiox10060967. PMID: 34208683 PMC8234027

[136] Heuer MA Pietrusko RG Morris RW Scheffler BJ . An analysis of worldwide safety experience with auranofin. J Rheumatol. (1985) 12:695–9. doi: 10.1007/bf03342628. PMID: 3932651

[137] Yu T Nie F-Q Zhang Q Yu S-K Zhang M-L Wang Q . Effects of methionine deficiency on B7H3-DAP12-CAR-T cells in the treatment of lung squamous cell carcinoma. Cell Death Dis. (2024) 15:12. doi: 10.1038/s41419-023-06376-w. PMID: 38182561 PMC10770166

[138] Ciudad MT Quevedo R Lamorte S Jin R Nzirorera N Koritzinsky M . Dabrafenib alters MDSC differentiation and function by activation of GCN2. Cancer Res Commun. (2024) 4:765–84. doi: 10.1158/2767-9764.CRC-23-0376. PMID: 38421883 PMC10936428

[139] GCN2 drives macrophage and MDSC function and immunosuppression in the tumor microenvironment | Science Immunology. doi: 10.1126/sciimmunol.aax8189 PMC7201901 31836669

[140] Dougé A Cueff G Keime C Carraro V Jousse C Rouzaire P . Transcriptomic analysis of human primary T cells after short-term leucine-deprivation and evaluation of kinase GCN2’s role in regulating differential gene expression. PloS One. (2025) 20:e0317505. doi: 10.1371/journal.pone.0317505. PMID: 39965008 PMC11835326

[141] Fleming S Tan S Mokoonlall M McQuillan A Yeh P Chuah H . First results from the Phase 1 study of APL-4098, an oral potent GCN2 inhibitor for the treatment of relapsed/refractory acute myeloid leukemia (R/R AML) and myelodysplastic syndromes (MDS)/AML. Blood. (2025) 146:6945. doi: 10.1182/blood-2025-6945

[142] Maiti A Post S DiNardo C Daver N Alvarado Valero Y Ohanian M . Phase 1b study of HC-7366 alone and in combination with venetoclax and azacitidine in relapsed/refractory acute myeloid leukemia. Blood. (2025) 146:1653. doi: 10.1182/blood-2025-1653

[143] Petralia F Ma W Yaron TM Caruso FP Tignor N Wang JM . Pan-cancer proteogenomics characterization of tumor immunity. Cell. (2024) 187:1255–1277.e27. doi: 10.1016/j.cell.2024.01.027. PMID: 38359819 PMC10988632

[144] Cieri N Camisa B Cocchiarella F Forcato M Oliveira G Provasi E . IL-7 and IL-15 instruct the generation of human memory stem T cells from naive precursors. Blood. (2013) 121:573–84. doi: 10.1182/blood-2012-05-431718. PMID: 23160470

[145] Weber EW Parker KR Sotillo E Lynn RC Anbunathan H Lattin J . Transient rest restores functionality in exhausted CAR-T cells through epigenetic remodeling. Science. (2021) 372:eaba1786. doi: 10.1126/science.aba1786. PMID: 33795428 PMC8049103

[146] Rappez L Stadler M Triana S Gathungu RM Ovchinnikova K Phapale P . SpaceM reveals metabolic states of single cells. Nat Methods. (2021) 18:799–805. doi: 10.1038/s41592-021-01198-0. PMID: 34226721 PMC7611214

[147] Delafiori J Shahraz M Eisenbarth A Hilsenstein V Drotleff B Bailoni A . HT SpaceM: A high-throughput and reproducible method for small-molecule single-cell metabolomics. Cell. (2025) 188:6028–6043.e11. doi: 10.1016/j.cell.2025.08.015. PMID: 40914160

[148] Medina CB Mehrotra P Arandjelovic S Perry JSA Guo Y Morioka S . Metabolites released from apoptotic cells act as tissue messengers. Nature. (2020) 580:130–5. doi: 10.1038/s41586-020-2121-3. PMID: 32238926 PMC7217709

[149] Perino A Schoonjans K . Metabolic Messengers: bile acids. Nat Metab. (2022) 4:416–23. doi: 10.1038/s42255-022-00559-z. PMID: 35338368

[150] Joung J Konermann S Gootenberg JS Abudayyeh OO Platt RJ Brigham MD . Genome-scale CRISPR-Cas9 knockout and transcriptional activation screening. Nat Protoc. (2017) 12:828–63. doi: 10.1038/nprot.2017.016. PMID: 28333914 PMC5526071

[151] Shifrut E Carnevale J Tobin V Roth TL Woo JM Bui CT . Genome-wide CRISPR screens in primary human T cells reveal key regulators of immune function. Cell. (2018) 175:1958–1971.e15. doi: 10.1016/j.cell.2018.10.024. PMID: 30449619 PMC6689405

[152] Heintzman DR Sinard RC Fisher EL Ye X Patterson AR Elasy JH . Subset-specific mitochondrial stress and DNA damage shape T cell responses to fever and inflammation. Sci Immunol. (2024) 9:eadp3475. doi: 10.1126/sciimmunol.adp3475. PMID: 39303018 PMC11607909

[153] Patterson AR Needle GA Sugiura A Jennings EQ Chi C Steiner KK . Functional overlap of inborn errors of immunity and metabolism genes defines T cell metabolic vulnerabilities. Sci Immunol. (2024) 9:eadh0368. doi: 10.1126/sciimmunol.adh0368. PMID: 39151020 PMC11590014

[154] Wang Z Diao Z Zhang Y Liu J Li Y Sun Z . Screening and mechanistic study of natural compounds that enhance T cell anti-tumor effects post-heat treatment. Front Immunol. (2025) 16:1537398. doi: 10.3389/fimmu.2025.1537398. PMID: 40213558 PMC11983556

[155] Wei Q Foyn H Landskron J Wang S Rye IH Skånland SS . Identification of a group of 9-amino-acridines that selectively downregulate regulatory T cell functions through FoxP3. iScience. (2025) 28:111931. doi: 10.1016/j.isci.2025.111931. PMID: 40034859 PMC11872463

[156] Maurya RP Prajapat MK Singh VP Roy M Todi R Bosak S . Serum malondialdehyde as a biomarker of oxidative stress in patients with primary ocular carcinoma: impact on response to chemotherapy. Clin Ophthalmol. (2021) 15:871–9. doi: 10.2147/OPTH.S287747. PMID: 33664564 PMC7924123

[157] Gernez E Deheul S Tard C Joncquel M Douillard C Grzych G . Plasma methionine and clinical severity in nitrous oxide consumption. Toxics. (2022) 11:12. doi: 10.3390/toxics11010012. PMID: 36668738 PMC9866764

[158] Satooka H Nakamura Y Hirata T . ROS-dependent SOCS3 upregulation disrupts regulatory T cell stability during autoimmune disease development. Redox Biol. (2025) 82:103590. doi: 10.1016/j.redox.2025.103590. PMID: 40090133 PMC11957609

[159] Keil M Sonner JK Lanz TV Oezen I Bunse T Bittner S . General control non-derepressible 2 (GCN2) in T cells controls disease progression of autoimmune neuroinflammation. J Neuroimmunology. (2016) 297:117–26. doi: 10.1016/j.jneuroim.2016.05.014. PMID: 27397084

[160] Ravishankar B Liu H Shinde R Chaudhary K Xiao W Bradley J . The amino acid sensor GCN2 inhibits inflammatory responses to apoptotic cells promoting tolerance and suppressing systemic autoimmunity. Proc Natl Acad Sci. (2015) 112:10774–9. doi: 10.1073/pnas.1504276112. PMID: 26261340 PMC4553766

[161] Kurniawan H FranChina DG Guerra L Bonetti L Baguet LS Grusdat M . Glutathione restricts serine metabolism to preserve regulatory T cell function. Cell Metab. (2020) 31:920–936.e7. doi: 10.1016/j.cmet.2020.03.004. PMID: 32213345 PMC7265172

